# Regulation of Multi-drug Resistance in hepatocellular carcinoma cells is TRPC6/Calcium Dependent

**DOI:** 10.1038/srep23269

**Published:** 2016-03-24

**Authors:** Liang Wen, Chao Liang, Enjiang Chen, Wei Chen, Feng Liang, Xiao Zhi, Tao Wei, Fei Xue, Guogang Li, Qi Yang, Weihua Gong, Xinhua Feng, Xueli Bai, Tingbo Liang

**Affiliations:** 1 Department of General Surgery, The Second Affiliated Hospital, Zhejiang University School of Medicine, Hangzhou, PR China; 2 Department of General Surgery, Ningbo Medical Treatment Center Lihuili Hospital, Ningbo, PR China; 3 Department of Neurosurgery Surgery, The Second Affiliated Hospital, Zhejiang University School of Medicine, Hangzhou, PR China; 4 Department of Hepatobiliary and Pancreatic Surgery, Henan Province People’s Hospital, Henan, PR China; 5 Center for Immunology and Microbial Disease,Albany Medical College, ME-205 47 New Scotland Ave., MC-151, Albany, New York, USA; 6 Life Sciences Institute and Innovation Center for Cell Signaling Network, Zhejiang University, Hangzhou, PR China; 7 Collaborative Innovation Center for Cancer Medicine, Zhejiang University, Hangzhou, PR China

## Abstract

Hepatocellular carcinoma (HCC) is notoriously refractory to chemotherapy because of its tendency to develop multi-drug resistance (MDR), whose various underlying mechanisms make it difficult to target. The calcium signalling pathway is associated with many cellular biological activities, and is also a critical player in cancer. However, its role in modulating tumour MDR remains unclear. In this study, stimulation by doxorubicin, hypoxia and ionizing radiation was used to induce MDR in HCC cells. A sustained aggregation of intracellular calcium was observed upon these stimuli, while inhibition of calcium signalling enhanced the cells’ sensitivity to various drugs by attenuating epithelial-mesenchymal transition (EMT), Hif1-α signalling and DNA damage repair. The effect of calcium signalling is mediated via transient receptor potential canonical 6 (TRPC6), a subtype of calcium-permeable channel. An *in vivo* xenograft model of HCC further confirmed that inhibiting TRPC6 enhanced the efficacy of doxorubicin. In addition, we deduced that STAT3 activation is a downstream signalling pathway in MDR. Collectively, this study demonstrated that the various mechanisms regulating MDR in HCC cells are calcium dependent through the TRPC6/calcium/STAT3 pathway. We propose that targeting TRPC6 in HCC may be a novel antineoplastic strategy, especially combined with chemotherapy.

Recently, the development of antineoplastic drugs has made great progress. However, their limited curative efficacy still remains a clinical obstacle, which is mainly ascribed to multi-drug resistance (MDR), induced by conventional drugs and also by new “targeted” drugs^1^,^2^. MDR also occurs in other situations, such as the hypoxic condition inside solid tumours^3^,^4^, making exploring the mechanisms of MDR a research hotspot. Numerous studies have revealed that MDR is associated with overexpression of certain drug efflux pumps^5^, epithelial mesenchymal transition (EMT)^6^,^7^, the hypoxia-inducible factor1-α (Hif1-α) signalling pathway^8^, DNA damage repair^1^,^9^,^10^,^11^, autophagy induction^12^ and epigenetic regulation^13^. Recently, some new factors such as cancer stem cell^14^, miRNAs^15^ and immunosuppressive microenvironment^16^, have also been implicated in MDR, rendering the mechanisms of MDR rather complicated.

Hepatocellular carcinoma (HCC) is an extremely malignant tumour with low sensitivity to chemotherapy, in part caused by MDR^17^. Several mechanisms govern MDR induction, among which drug efflux pump, EMT, Hif1-α signalling and DNA damage repair play vital roles in the chemo-resistance of HCC^16^,^18^,^19^,^20^. It is frequently observed that one mechanism cannot be fully responsible for acquired chemo-resistance to drugs; therefore, a strategy targeting one mechanism alone is always poorly effective. Studies on the relationships between various MDR mechanisms are scarce. Therefore, identifying a common key signalling pathway is a promising approach to improve the efficacy of chemotherapy. Herein, HCC cells were treated by stimulation with doxorubicin, hypoxia and ionizing radiation, representing three models of MDR, to identify for possible common signalling events related to crucial MDR mechanisms.

Intracellular calcium is a versatile second messenger that is involved in many physical and pathological processes. The calcium signalling pathway plays a vital role in tumour cells, via apoptosis, proliferation, invasion and metastasis^21^. Some studies demonstrated that MDR-relevant mechanisms of EMT, hypoxia-induced Hif1-α signalling pathway and DNA damage repair are closely related to intracellular calcium. In breast cancer cells, various stimuli-induced EMT are dependent on changes in non-stimulated store-operated calcium entry^22^,^23^, partly via calcium channel TRPM7^24^. In addition, calcium participates in enhanced Hif-1 transcriptional activity in cells under hypoxia^25^,^26^. Calcium is also an important co-factor in genotoxic stress from poly polymerase-1 hyperactivation after reactive oxygen species (ROS)-induced DNA damage-related alterations in cellular metabolism and DNA repair^27^. However, there have been few studies on the common interactions between intracellular calcium and enhanced drug resistance driven by these mechanisms.

Intracellular calcium homeostasis is regulated by the calcium channels/pumps, mostly in the cell membrane and endoplasmic reticulum. In oncology, altered expressions of specific calcium channels and pumps are characteristic features of certain cancers^28^ and have been studied thoroughly in recent years. Interestingly, among all the calcium channels/pumps, transient receptor potential (TRP) calcium channels have come to our attention because of their wide roles in malignant behaviours of cancer cells, including proliferation, migration and invasiveness^28^,^29^. Indeed, it was reported that TRP canonical 5 (TRPC5) is essential for P-glycoprotein (P-gp) induction in drug-resistant cancer cells^30^. Even so, there are few studies connecting the role of TRP channels with chemotherapy resistance. Specifically in liver cancer, both TRP canonical 6 (TRPC6) and TRP canonical 1 (TRPC1) are associated with cell proliferation^31^. TRPC6 is poorly expressed in normal hepatocytes, but highly expressed in liver carcinoma samples^32^. However, the role of TRP calcium channels in chemo-resistance through calcium is seldom studied and still remains unclear. Hence, in this study, we explored the roles of intracellular calcium on various MDR relevant mechanisms, and further investigated its upstream TRP calcium channel and the common downstream regulator in HCC cells.

## Results

### Stimulation by doxorubicin, hypoxia or ionizing radiation enhance HCC cells’ resistance to multiple drugs

**Figure 1:**
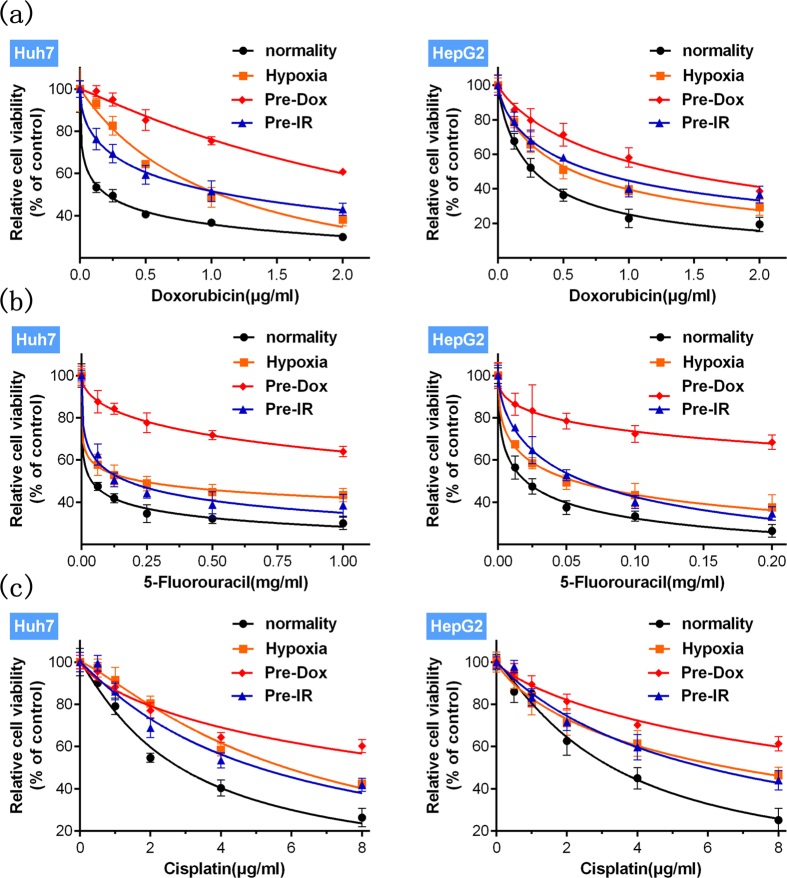
Enhanced multi-drug resistance induced by various stimuli. Stimuli with doxorubicin (Pre-Dox) (red), hypoxia (orange) and ionizing radiation (Pre-IR) (blue) all significantly (p < 0.05, stimuli group *vs*. normality group) increased the relative cell viability (Mean ± SD) (n = 6) of Huh7 and HepG2 cells, treated by various concentrations of (**a**) doxorubicin, (**b**) 5-fluorouracil or (**c**) cisplatin. Best-fit lines are presented. Statistical significances were assessed using one-way ANOVA with Bonferroni’s post-tests.

To study MDR relevant mechanisms in HCC, cells were separately treated with doxorubicin, hypoxia and ionizing radiation to build three acquired MDR models. The optimal dose and duration of the various stimuli were determined according to previous reports and our preliminary experiments (see Supplementary Fig. S1) to ensure stable MDR induction. After various stimuli, the altered cells’ resistance to multiple drugs was assessed by drug sensitivity assays using three widely used drugs. Doxorubicin, hypoxia and ionizing radiation all significantly decreased the cells’ sensitivity to doxorubicin, 5-fluorouracil and cisplatin (Fig. 1). The mean IC_50_ (half maximal inhibitory concentration) values of multiple drugs in HCC cells, determined by full-range concentrations of the various drugs, were significantly elevated compared with groups not exposed to stimuli (Table 1), which was consistent with the results shown in Fig. 1. Thus, stimulation using doxorubicin, hypoxia or ionizing radiation successfully induced MDR in HCC cells.

### Sustained aggregation of intracellular calcium participates during various stimuli

**Figure 2:**
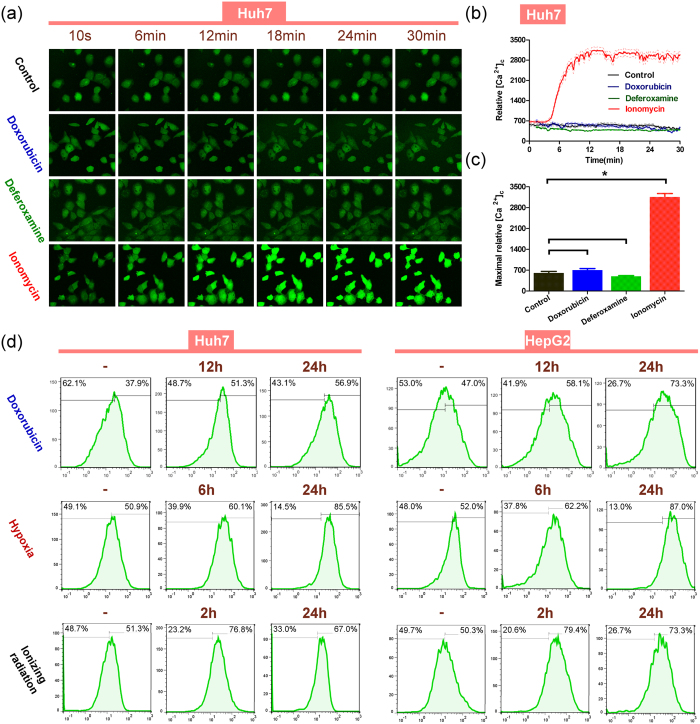
Various stimuli produce a significantly long-term and sustained, but not immediate, increase in cytosolic calcium. (**a**) Dynamic monitoring of [Ca^2+^]_c_ immunofluorescence within 30 min after adding doxorubicin (0.2 μg/mL), deferoxamine (100 μM), ionomycin (2 μg/mL) or HBSS (control group) to Huh7 cells incubated with Fluo4-AM calcium indicator. Ionomycin, known as an ionophore Ca^2+^, was used as a positive control to increase [Ca^2+^]_c_ markedly. (**b**) Changes in mean cytosolic calcium fluorescence values of Huh7 cells (n = 11–18) treated by doxorubicin (0.2 μg/mL) (blue), deferoxamine (100 μM) (green) and ionomycin (2 μg/mL) (red) are shown, with the mean lines (solid) and 95% CI lines (dashed). Reagents were added at the 3 min. time point. (**c**) The maximum calcium fluorescence values (mean ± SD) were calculated after adding doxorubicin (blue), deferoxamine (green) and ionomycin (red) within 30 min, respectively. (*p < 0.05, each treatment group *vs*. control group). (**d**) Cells were treated by doxorubicin (0.2 μg/mL), hypoxia (1% O_2_) and ionizing radiation (10Gy) and the cytosolic calcium immunofluorescence was measured by flow cytometry. Vertical split lines were set at the same fluorescence intensity level and right proportion of cell numbers was calculated. Statistical significances were assessed using Student’s T-Test.

We next explored the effects of these three stimuli on intracellular cytosolic free calcium ([Ca^2+^]_c_) in HCC cells. Deferoxamine (DFO), an Fe chelating agent that was reported as an Hif1-α and Hif1-α-dependent proteins inducer^33^,^34^, was applied as a hypoxia analogue. The ability of certain stimuli (e.g., mechanical scratch, growth factors) to increase [Ca^2+^]_c_ is always transient and sometimes shows a spatial and temporal aspect^35^. However, doxorubicin or DFO failed to induce a rapid [Ca^2+^]_c_ aggregation or evident fluctuation, compared with the control group (Fig. 2a and see Supplementary Video 1, 2, 3 and 4). Quantitative analysis of [Ca^2+^]_c_ aggregation (Fig. 2b) and the maximal contents of [Ca^2+^]_c_ (Fig. 2c) during these stimuli in Huh7 cells showed the same result. The same phenomenon was also observed in HepG2 cells (see Supplementary Fig. S2 and Video 5, 6, 7 and 8).

We then tested long-term [Ca^2+^]_c_ changes in HCC cells. The [Ca^2+^]_c_ fluorescence intensity was measured (Fig. 2d) after various stimuli within 24 h. Separated by the vertical split line, cells with relatively more [Ca^2+^]_c_ were defined as [Ca^2+^]_c_ positive cells. The percentage of [Ca^2+^]_c_ positive cells among those treated by doxorubicin gradually increased at 12 h (51.3%) and 24 h (56.9%), compared with the untreated group (37.9%). In HepG2 cells, the percentage was 47.0% (control), 58.1% (12 h) and 73.3% (24 h). Under hypoxia, the percentage of [Ca^2+^]_c_ positive cells also increased at 6 h (60.1% (Huh7) and 62.2% (HepG2)) and 24 h (85.5% (Huh7) and 87.0% (HepG2)), compared with the untreated groups (50.9% (Huh7) and 52.0% (HepG2)). Similarly, the percentage of [Ca^2+^]_c_ positive cells increased both 2 h (76.8% (Huh7) and 79.4% (HepG2)) and 24 h (67.0% (Huh7) and 73.3% (HepG2)) after exposure to ionizing radiation, compared with the untreated groups (51.3% (Huh7) and 50.3% (HepG2)). Repeated experiments gave the same results. The relative percentages of [Ca^2+^]_c_ positive cells were calculated and analysed (see Supplementary Fig. S3). These results demonstrated that under long-term stimulation by doxorubicin, hypoxia or a long time after ionizing radiation, HCC cells could maintain a sustained aggregation of intracellular calcium, indicating that long-term elevation of [Ca^2+^]_c_ is more likely to play a significant role in cells’ response to various stimuli.

### Intracellular calcium chelation inhibits stimuli-induced MDR and its relevant mechanisms

**Figure 3:**
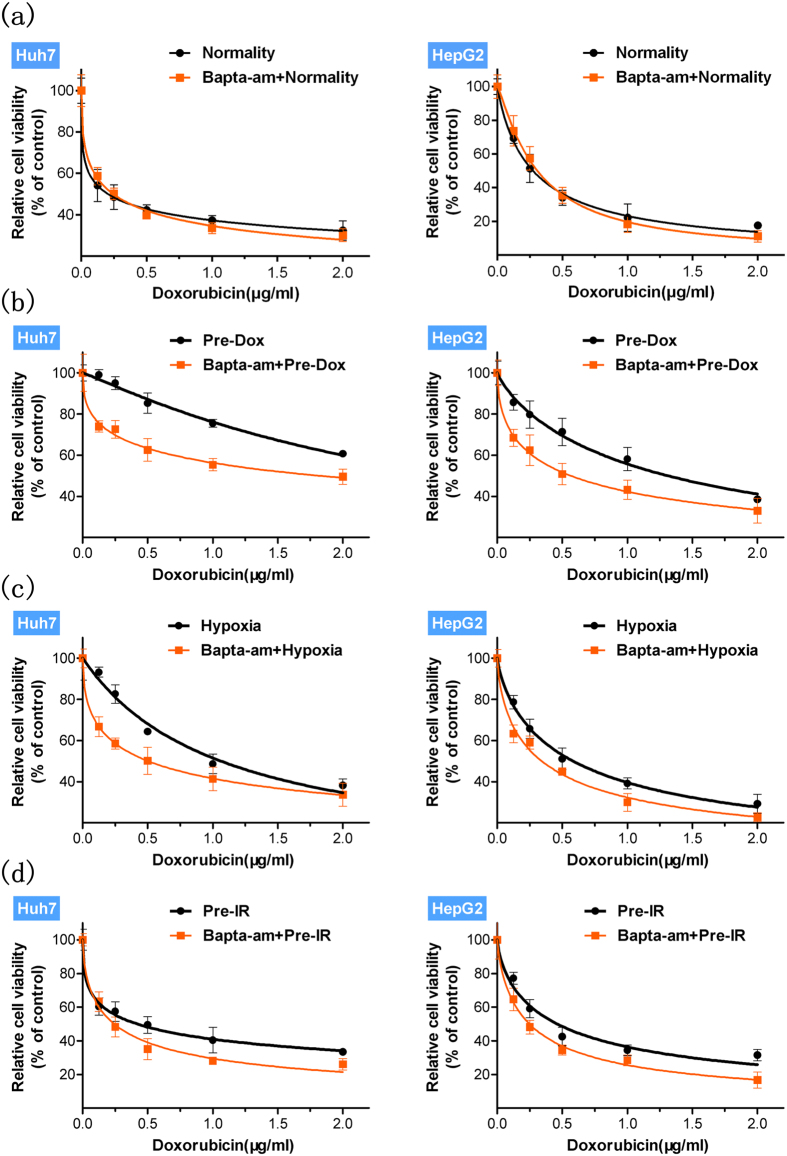
Calcium chelation (**a**) fails (p > 0.05) to affecte the drug sensitivity of unstimulated cells (normality) to doxorubicin but significantly (p < 0.05) attenuates the enhancement of HCC cells’ resistance to doxorubicin by stimuli with (**b**) doxorubicin (Pre-Dox), (**c**) hypoxia and (**d**) ionizing radiation (Pre-IR). Relative cell viability (Mean ± SD) (n = 6) was calculated for control (black) and calcium chelation (orange) groups and best-fit lines are presented. Statistical significances were assessed using one-way ANOVA with Bonferroni’s post-tests.

Based on various observed simultaneous stimuli-induced MDR and [Ca^2+^]_c_ elevation, we investigated whether calcium signalling participates in MDR. Before applying various stimuli, HCC cells were pretreated with the intracellular calcium chelator, BAPTA-AM, at a maximal dose (10 μM), which has been shown to be not significantly toxic to normal HCC cells over the duration of the stimuli (see Supplementary Fig. S4). And calcium chelation affected the drug sensitivity of unstimulated cells to drugs very slightly (Fig. 3a and see Supplementary Fig. S5). However, after stimulation with doxorubicin, hypoxia or ionizing radiation, we found intracellular calcium chelation significantly attenuated the cells’ resistance to various drugs (Fig. 3(b–d) and see Supplementary Fig. S5). And the mean IC_50_ values of drugs were presented to further assess the altered drug sensitivity of HCC cells with calcium chelation (see Supplementary Table S1). Taken together these data indicated that intracellular calcium is involved in the MDR induced by various stimuli in HCC cells.

Upregulation of ABC transporters and induction of EMT are mainly responsible for doxorubicin-induced MDR, which were well studied in HCC in our previous published studies^36^,^37^. Preliminary experiments revealed that calcium chelation failed to inhibit doxorubicin-induced upregulations of ABC transporters (see Supplementary Fig. S6) but not EMT (supported by the following data); therefore, EMT was selected in this study. Hif1-α signalling under hypoxia and DNA damage repair in radiation treatment are widely regarded as the crucial responding mechanisms^15^. Specifically, Twist and H2A.X play crucial roles in doxorubicin-induced EMT^6^ and ionizing radiation-induced DNA damage repair^38^, respectively. Indeed, we confirmed that the mechanisms of EMT, Hif1-α signalling and DNA damage repair play roles in regulating MDR induced by doxorubicin, hypoxia and ionizing radiation, using short interfering RNA (siRNA) downregulation of *Twist*, *Hif1-α* and *H2A.X* (see Supplementary Fig. S7), respectively. That was the rationale for selecting EMT, Hif1-α signalling and DNA damage repair as the model mechanisms to investigate MDR corresponding to various stimuli in this study.

**Figure 4:**
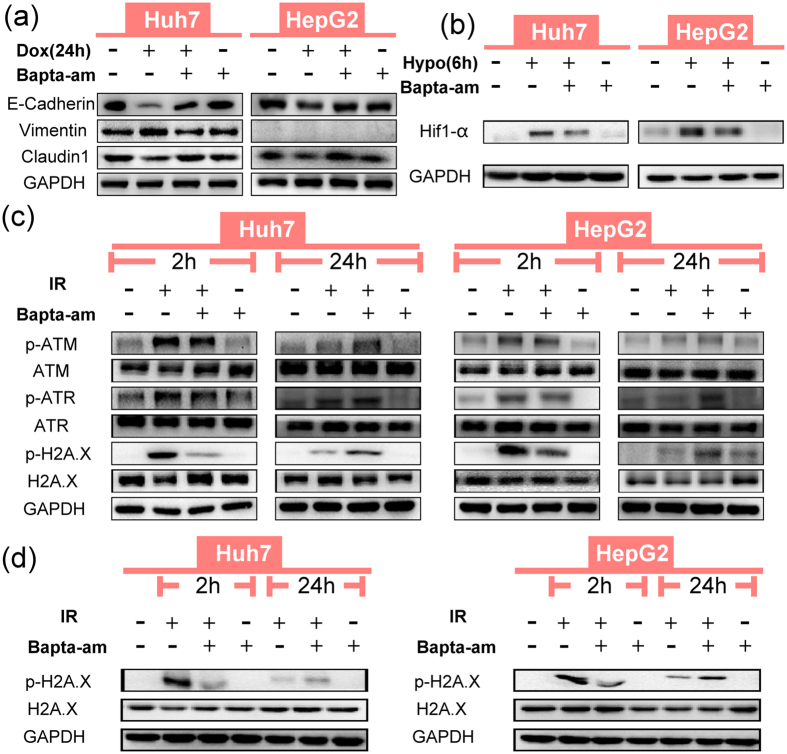
Calcium chelation inhibits EMT, Hif1-α signalling and DNA damage repair, induced by doxorubicin, hypoxia and ionizing radiation respectively, in Huh7 and HepG2 cells. (**a**) Expression of EMT associated proteins E-Cadherin, Claudin1 and Vimentin (HepG2 cells express extremely low Vimentin) with or without BAPTA-AM (10 μM). (**b**) Expression of Hif1-α under hypoxia, with or without BAPTA-AM (10 μM). (**c**) Expression of DNA damage repair proteins, p-ATM, p-ATR and p-H2A.X 2 h and 24 h after IR (10Gy), with or without BAPTA-AM (10 μM). (**d**) Comparison of p-H2A.X expressions 2 h and 24 h after IR (10Gy), with or without BAPTA-AM (10 μM).

The role of intracellular calcium in regulating these three mechanisms was then examined. We found that doxorubicin reduced the expression of E-Cadherin and Claudin1 and upregulated Vimentin, while calcium chelation reversed the doxorubicin-induced expression changes of EMT markers (Fig. 4a). In addition, calcium chelation also reversed the spindle-like morphology of HCC cells induced by doxorubicin to a grape-like one (see Supplementary Fig. S8a). For the hypoxia-induced Hif1-α signalling pathway, calcium chelation blocked the expression of hypoxia-induced Hif1-α strongly (Fig. 4b). As for the third mechanism, to assess DNA damage repair activation induced by ionizing radiation, the phosphorylation of H2A.X, ATM and ATR, were chosen as classical proteins. At 2 h after ionizing radiation treatment, calcium chelation decreased the phosphorylation of ATM, ATR and H2A.X in HCC cells. By contrast, 24 h after ionizing radiation, groups with calcium chelation showed a relatively higher expression of p-ATM, p-ATR and p-H2A.X, compared with the groups treated with ionizing radiation alone (Fig. 4c). We then compared the relative expression levels of p-H2A.X at the 2 h and 24 h time points simultaneously to assess the overall influence of calcium chelation. And the result showed that p-H2A.X expression at 24 h after ionizing radiation, regardless of calcium chelation, was much lower than that at 2 h (Fig. 4d), indicating that the peak of DNA damage repair activation appears at an early stage and DNA damage repair was more affected by calcium chelation at the 2 h time point. In addition, a comet assay revealed that calcium chelation significantly increased the severity of DNA damage after ionizing radiation (see Supplementary Fig. S8(b,c)). Thus, on the whole, calcium chelation efficiently inhibited the cells’ capacity for DNA damage repair within 24 h after ionizing radiation. Taking into account that the marked expression of p-H2A.X 2 h after ionizing radiation was more responsible for DNA damage repair, the expressions of DNA damage repair proteins at 2 h time point after ionizing radiation were tested in the subsequent MDR relevant studies.

In summary, these findings demonstrated that in HCC cells, the MDR-related mechanisms, EMT, Hif1-α signalling and DNA damage repair, are all calcium dependent.

### TRPC6 modulates calcium-dependent MDR

**Figure 5:**
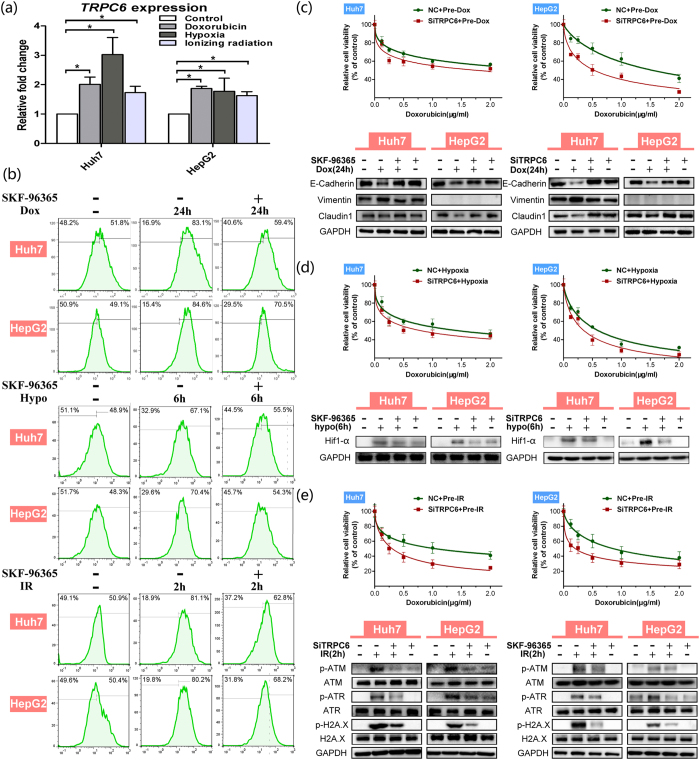
The TRPC6 channel plays a vital role in various mechanisms to regulate MDR via calcium in Huh7 and HepG2 cells. (**a**) Relative expression of TRPC6 mRNA (Mean ± SD) (n ≥ 3) after treatment with doxorubicin (0.2 μg/mL) for 24 h, hypoxia (1% O_2_) for 24 h or ionizing radiation (10 Gy) (24 h later), compared with control groups, respectively, in Huh7 and HepG2 cells (*p < 0.05, treatment group *vs*. control group). (**b**) Stimuli with doxorubicin (Dox), hypoxia (Hypo) or ionizing radiation (IR), with or without TRPC6 inhibitor SKF-96365 (10 μM) in HCC cells. The [Ca^2+^]_c_ immunofluorescence was measured by flow cytometry. Vertical spilt lines were set at the same fluorescence intensity level and the right proportion of cells was calculated in HCC cells. SKF-96365 (10 μM) partly reduced the positive [Ca^2+^]_c_ cells ratio after cells treated by doxorubicin for 24 h, hypoxia for 6 h or ionizing radiation (2 h later). TRPC6 interference significantly (p < 0.05) attenuated the enhancement of HCC cells’ resistance to doxorubicin by stimuli of doxorubicin (**c**), hypoxia (**d**), or ionizing radiation (**e**). Relative cell viability (Mean ± SD) (n = 6) was calculated for negative control (NC) (green) groups and TRPC6 siRNA (siTRPC6) (red) groups and best-fit lines are presented. Significant differences were assessed using one-way ANOVA with Bonferroni’s post-tests (p < 0.05, siTRPC6 group *vs*. NC group). Western blotting demonstrating the expressions of E-Cadherin, Claudin1 and Vimentin in doxorubicin treatment for 24 h (**c**), expression of Hif1-α under hypoxia for 6 h (**d**), and expression of DNA damage repair proteins p-ATM, p-ATR and p-H2A.X 2 h after ionizing radiation treatment (**e**) in NC and siTRPC6 HCC cells. Statistical significances were assessed using Student’s T-Test.

We next explored the potential calcium channel/pump responsible for intracellular calcium aggregation to regulate of MDR. Accumulating evidence has shown that the TRP channels are intimately associated with tumour behaviours. Huh7 and HepG2 cells mainly express TRPC1 and TRPC6^32^, which were considered first in our study. We found that in HCC cells, stimulation by doxorubicin, hypoxia and ionizing radiation significantly increased the mRNA expression of *TRPC6* (Fig. 5a) but not of *TRPC1* (data not shown). In addition, in HCC tissues, we found that *TRPC6* expression is much higher in tumour tissues than in pericancerous tissues and is markedly related to a higher TNM classification (data not shown), suggesting that TRPC6 is closely associated with HCC’s malignant behaviours. Based on this, we hypothesized TRPC6 might also be responsible for HCC’s acquired MDR via calcium signalling.

To explore the function of TRPC6 in regulating long-term [Ca^2+^]_c_ aggregation, the specific TRPC6 inhibitor, SKF-96365, was used. The results showed that SKF-96365 significantly reduced the number of [Ca^2+^]_c_ positive cells under any stimulus in HCC cells (Fig. 5b). This was confirmed by quantitative analysis (see Supplementary Fig. S9). Subsequently, both the TRPC6 inhibitor and a TRPC6 interfering siRNA (siTRPC6) were used to further clarify the role of TRPC6 in MDR. The efficiency of TRPC6 interference is shown in Supplementary Fig. S10. After TRPC6 interference, HCC cells showed significant attenuation of drug resistance under all three stimuli, as seen in Fig. 5(c–e) and Supplementary Fig. S11. TRPC6 inhibition could reverse doxorubicin-induced EMT (Fig. 5c), block hypoxia-induced Hif1-α expression (Fig. 5d) and inhibit DNA damage repair at 2 h after ionizing radiation (Fig. 5e).

These results demonstrated that the calcium channel TRPC6 plays a vital role in the sustained aggregation of [Ca^2+^]_c_ under various stimuli, which in turn is directly involved in regulating EMT, Hif1-α signalling and DNA damage repair.

### STAT3 activation mediates calcium dependent MDR

**Figure 6:**
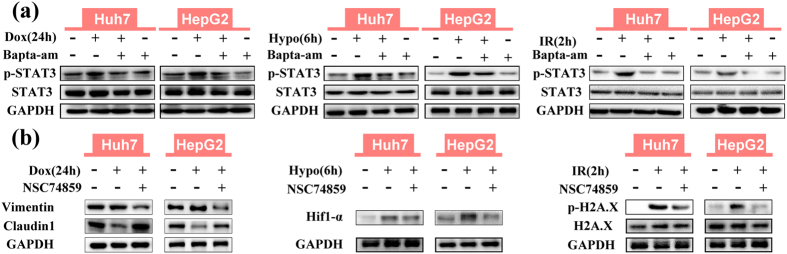
Phosphorylation of STAT3 (Tyr705) is blocked by intracellular calcium chelation and STAT3 inhibitor NSC74859 (100 μM) attenuates various MDR-related mechanisms in HCC cells. (**a**) Western blotting demonstrating the expression of p-STAT3 induced by doxorubicin (0.2 μg/mL) (Dox) for 24 h, hypoxia (1% O_2_) (Hypo) for 6 h and ionizing radiation (10 Gy) (IR) (2 h later), with or without BAPTA-AM (10 μM). (**b**) Western blotting demonstrating the expressions of Claudin1 and Vimentin after doxorubicin treatment, Hif1-α under hypoxia and p-H2A.X 2 h after ionizing radiation with or without NSC74859 (100 μM).

The common bridge between calcium signalling and the downstream mechanisms remains unclear. Interestingly, the mechanisms of EMT, Hif1-α signalling and DNA damage repair were all reported to be regulated by phosphorylation of upstream proteins, such as Erk, AKT and STAT3^39^,^40^,^41^,^42^,^43^, which were reported to promote drug resistance in tumours. Based on this, calcium dependent calmodulin phosphorylase targeted Erk1/2, AKT and STAT3 were hypothesized to connect calcium and MDR-relevant mechanisms^21^,^24^. 2 h after various stimuli in HCC cells, calcium chelation markedly increased the expression of p-Erk1/2 and p-AKT. In contrast, calcium chelation decreased p-STAT3 expression (Fig. 6a and see Supplementary Fig. S12), which was consistent with the attenuated drug resistance. Furthermore, both calcium chelation and TRPC6 interference significantly inhibited the increased p-STAT3 expression by all stimuli at 24 h, 6 h and 2 h for doxorubicin, hypoxia and ionizing radiation (Fig. 6a and see Supplementary Fig. S13), respectively, indicating that STAT3 activation acts as a downstream regulator in TRPC6/calcium signalling.

Furthermore, STAT3 inhibition by NSC74859^44^,^45^ also significantly attenuated various stimuli-induced resistance to doxorubicin (see Supplementary Fig. S14). In terms of the mechanism, NSC74859 reversed doxorubicin-induced EMT, blocked hypoxia-induced Hif1-α expression and inhibited DNA damage repair at 2 h after ionizing radiation, which were similar to the effects of calcium chelation (Fig. 6b). Taken together, these results showed that calcium-regulated STAT3 activation acts as the downstream regulator to mediate EMT, Hif1-α signalling and DNA damage repair in HCC cells.

### TRPC6 silencing enhances the efficacy of doxorubicin for HCC *in vivo*

**Figure 7:**
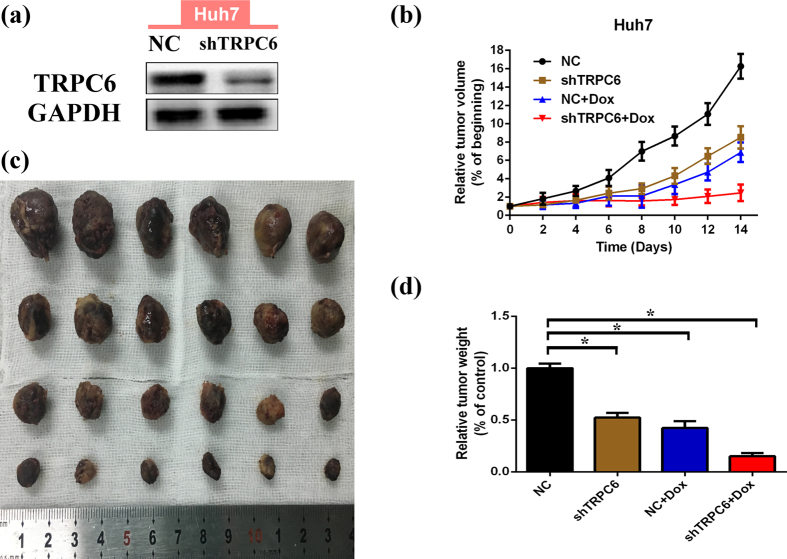
TRPC6 silencing enhances the curative efficacy of doxorubicin in Huh7 cells xenografts in nude mice. (**a**) TRPC6 protein expression after Huh7 cells were infected with LV-NC or LV-shTRPC6 virus and cultured until for subcutaneous injection. (**b**) Tumour xenografts volumes develop since doxorubicin treatments began. Groups of negative control (NC) cells (black), NC cells treated by doxorubicin (blue), TRPC6 silencing (shTRPC6) cells (brown) and shTRPC6 cells treated by doxorubicin (red). Relative tumour volumes (% of initial volume when treatments began) are presented as the mean ± SD (n = 6). (c) The tumour xenografts were presented 2 weeks after doxorubicin treatment. (**d**) Tumour xenografts were finally weighted. Groups of negative control (NC) cells (black), NC cells treated by doxorubicin (blue), TRPC6 silencing (shTRPC6) cells (brown) and shTRPC6 cells treated by doxorubicin (red). Relative tumour weights (% of “NC” group) are presented as the mean ± SD (n = 6). Statistical significances were assessed using Student’s T-Test (*p < 0.05, each groups *vs*. “NC” group).

To explore the *in vivo* role of TRPC6 in the efficacy of doxorubicin for HCC, the stable TRPC6-silenced Huh7 cells were obtained using lentiviral infection with a short hairpin RNA-based vector. The xenograft models were established via subcutaneous injection of cells, and tumour growth was monitored following administration of doxorubicin every other day. Two weeks after implantation, mice were sacrificed and the tumours were dissected and weighted. We found that TRPC6 -silenced cells grew much more slowly than normal cells and were more sensitive to doxorubicin (Fig. 7(a–c)). The calculated coefficient index (CI), as described previously^46^, was less than 1 (CI = 0.682), indicating a synergistic effect of targeting TRPC6 in combination with doxorubicin *in vivo*.

## Discussion

HCC is a deadly disease that shows insensitivity to chemotherapy because of easily acquired MDR in many circumstances. Previous studies aiming to overcome MDR or chemo-resistance were restricted to single-target research, which usually resulted in limited curative efficacy. Among the potential key signalling pathways, calcium signalling has been shown to regulate the MDR relevant mechanisms of EMT, Hif1-α signalling and DNA damage repair simultaneously. Moreover, we also verified that the promising calcium channel TRPC6 is responsible for calcium aggregation and MDR induction. Our study showed, for the first time, that calcium channel TRPC6-mediated intracellular calcium aggregation plays a vital role in various mechanisms that regulate MDR in HCC cells.

In this study, we determined that the MDR relevant mechanisms of EMT, Hif1-α signalling and DNA damage repair were all calcium aggregation-dependent in HCC cells. Previous studies revealed that powerful calcium signalling participates in these mechanisms in other circumstances. EGF-induced EMT in breast cancer cells is calcium dependent^24^. In MCF-7 cells, β-lapachone-induced p-H2A.X foci formation is regulated by calcium^47^. Specifically in our ionizing radiation-induced MDR model, though on the whole calcium chelation could inhibit the DNA damage repair, its effect seemed to be invalid at the later stage (24 h), suggesting the potential of other existed compensatory signaling pathways to regulate DNA damage repair, as the respond to the long-term calcium elimination. On the other hand, in our study, preliminary experiments showed that intracellular calcium chelation failed to attenuate the upregulation of ABC transporters. L-type calcium channel blockers, such as verapamil, were found to antagonize MDR1 expression, which could not be reversed by simple intracellular calcium chelation^48^. Another limitation is that other MDR-relevant mechanisms have not been explored to further determine the interactions of calcium with MDR. Thus, intracellular calcium signalling is very complicated and it was not possible to determine whether it mediates all MDR-relevant mechanisms.

The TRP family of calcium channels are mainly involved in the store-operated Ca^2+^ entry (SOCE)^49^ and non-capacitative Ca^2+^ entry (NCCE)^50^,^51^. Under different stimuli, both TRP channels-induced SOCE and NCCE result in a transient intracellular calcium aggregation and initiated a rapid alteration to the calcium signalling pathway. However, the rapid intracellular calcium accumulation induced by 1-oleoy-2-acetyl-glycerol (OAG), which is thought to be involved in NCCE^52^, was not affected by overexpression of TRPC6^32^. Therefore, the comprehensive role of TRP channels still remains obscure. Recent reports indicated that TRPC6 also regulates the sustained elevation of basal calcium to influence tumour malignant behaviours. Under hypoxia, TRPC6 causes a sustained elevation of intracellular calcium that is coupled with the development of malignant glioblastoma multiforme cells^29^. In hepatic stellate cells under hypoxia, the expression of TRPC6 causes a sustained elevation of intracellular calcium, which activates the synthesis of extracellular matrix proteins^53^. In our study, the sustained elevation of intracellular free calcium was also regulated by TRPC6 overexpression, not only under hypoxia, but also under stimuli of doxorubicin and ionizing radiation. Based on this evidence, we concluded that by modulating sustained intracellular calcium aggregation, the overexpression of TRPC6 has crucially important effect on the adaptive behaviours of tumours.

*In vitro*, blocking TRPC6 by either siRNA or SKF-96365 attenuated MDR induced by various stimuli. *In vivo*, targeting TRPC6 showed a synergistic antitumor effect when combined with doxorubicin. Meanwhile, a significant inhibitory role of TRPC6 on HCC cells proliferation was also observed, which was consistent with previous reports^29^,^32^. Thus, the functions of TRPC6 are extensive and not only limited to MDR amelioration.

The downstream signalling pathways regulated by intracellular free calcium are a complicated issue. It is related to disturbing the balance of various kinases/phosphorylases, the activation of calcium binding proteins, the transcription of genes and even alteration of the calcium current by calcium sensitive channels or pumps^21^. Epigenetic regulation, including DNA methylation, protein phosphorylation and histone modification^15^,^54^, have been reported to aid cancer cells to obtain adaptive MDR. In our study, the common calcium signalling downstream event was further explored, and STAT3 was identified. The pathological activation of STAT3 to induce EMT and Hif1-α in human carcinomas has been widely studied^55^,^56^. The cdk5-STAT3 oncogenic pathway plays an important role in the expression of DNA repair genes^57^. Taken together, we demonstrated that activation of STAT3 acts as a bridge to connect calcium and various MDR-related mechanisms.

In summary, our study identified the pivotal role of calcium in mediating mechanisms of EMT, Hif1-α signalling and DNA damage repair to obtain MDR in HCC cells, which were attributed to the sustained accumulation of intracellular free calcium by TRPC6 overexpression. Considering the significant roles of calcium in physical and pathological processes, and the complexity of calcium signalling, simply altering calcium signalling would not render cancer cells more vulnerable and could even have unpredictable systemic consequences^28^. Therefore, we propose that targeting TRPC6 in HCC might represent a possible novel antineoplastic strategy, especially when combined with chemotherapy.

## Methods

### Cell culture and reagents

Human HCC cell lines (Huh7 and HepG2, the Shanghai Institution for Biological Science, Shanghai, China) were cultured in DMEM medium (Gibco, Carlsbad, CA, USA) with 10% fetal bovine serum (FBS) (Gibco) and 1% penicillin/streptomycin (Sigma, St. Louis, MO, USA). Cells were normally cultured in a humidified incubator at a constant temperature of 37 °C and 5% CO_2_. Doxorubicin, 5-fluorouracil, cisplatin, BAPTA-AM, SKF-96365, DFO and ionomycin were all purchased from Sigma. Fluo4-AM and PluronicF-127 were purchased from Invitrogen (Carlsbad, CA, USA). NSC74859 was purchased from Merck KgaA (Darmstadt, Germany). The stock solutions were prepared in water, PBS or dimethyl sulphoxide (DMSO), as required and stored at −20 °C. Once diluted, the final concentration of DMSO was less than 0.5% to avoid toxicity to cells. To induce EMT with doxorubicin stimuli, HCC cells were treated with 0.2 μg/mL doxorubicin for 24 h. To induce expression of Hif1-α by hypoxia, cells were cultured in a low oxygen incubator with 1% O_2_, 5% CO_2_ and 94% N_2_ for 6 h. Ionizing radiation (X-ray) was produced by an electron accelerator (Siemens, Germany) in the author’s hospital. To obtain DNA damage repair, cells were transiently treated with 10Gy ionizing radiation at 0.5Gy/min and then after 2 h, the cells were normally cultured and the subsequent drug sensitivity assays were performed. For the chelation of intracellular calcium, cells were loaded with 10 μM BAPTA-AM for 1 h before various stimuli. To specifically inhibit TRPC6 function and STAT3 activation respectively, SKF-96365 (10 μM) and NSC74859 (100 μM) were added into the culture medium for the duration of stimulation.

### Relative cell viability assays by CCK8

Relative cell viability of drug sensitivity assays was measured by using the Cell Counting Kit-8 (CCK8) kit (Dojindo, Kumamoto, Japan). 3.5 × 10^3^ cells per well (100 μL media) were seeded into 96 well microplates. After incubation for 12 h, cells were then treated by various stimuli as described above. Cells were then treated with various drugs (doxorubicin, 5-fluorouracil and cisplatin) for 48 h; 10 μL CCK8 solution was added into each well. After incubation for 3 h, the OD values were measured at 450 nm using an MRX II microplate reader (Dynex, Chantilly, VA, Canada). The relative cell viability was determined as a percentage of the untreated control groups. In addition, the IC_50_ was calculated by the equation: 100a% = 100/(1 + 10^log[drug]−logIC50^), with the relative cell viability of 100a% and the drug concentration as [drug].

### Intracellular calcium measurement

#### Laser confocal microscopy (LSCM)

For dynamic monitoring of the rapid [Ca^2+^]_c_ fluctuation, fluo4-AM was chosen as the intracellular free calcium probe. HCC cells were seeded in 33cm confocal specific dishes and grown to 30–70% confluence. The cells were loaded with 5 μM Fluo4-AM in HBSS (Gibco) containing 0.05% PluronicF-127 at 37 °C for 40 min and then washed three times by HBSS to remove the extra Fluo4-AM. To record the [Ca^2+^]_c_ fluorescence, LSCM was performed using an Olympus laser confocal system (Olympus, IX81-FV1000) with continuous image recording, at a magnification of 40× at 2.343 s intervals, for 30 min. The parameters of LSCM were set as an excitation wavelength of 488 nm and an emission wavelength of 516 nm. Administration of doxorubicin, DFO or ionomycin was performed 3 min after recording began. The relative [Ca^2+^]_c_ content was presented as the calcium fluorescence intensity.

#### Fluorescence activated cell sorting (FACS)

To measure the intracellular calcium intensity in HCC cells treated with doxorubicin, hypoxia, ionizing radiation, Fluo4-AM loading was performed as above. The calcium fluorescence intensity was then measured in the fluorescence channel FITC in FACS analysis. The FACS assay was performed using a flow cytometer (FACS Canto II, BD, USA).

### Optical microscope observation

HCC cells were seeded in 6-well plates until confluence and then treated with doxorubicin (0.2 μg/mL) for 48 h with or without calcium chelation (10 μM BAPTA-AM for 1 h). Images were recorded under a Zeiss Axio Observer optical microscope using a 40× objective.

### Comet assay/Single-cell gel electrophoresis and analysis

The degree of DNA damage was measured and analysed using single-cell gel electrophoresis (Comet assay) and a Comet assay kit (GMS, USA). The experiment was performed according to the manufacturer’s instructions. After receiving ionizing radiation (10Gy) with or without calcium chelation (10 μM BAPTA-AM for 1h), cells were harvested in a density of 1 × 10^6^ cells/mL in HBSS and mixed with liquefied agarose at a 1:9 (v/v) ratio. 100 μL of the mixture was immediately transferred to agarose-coated slides and lysed at 4 °C in the dark for 2 h. The slides were then transferred into the alkaline solution containing 0.6 mM Na-EDTA and 0.18 mM NaOH (pH = 13) at room temperature for 30 min to loosen double-stranded DNA. Later, the slides were removed from the above alkaline solution and electrophoresed at 25 volts in the electrophoresis apparatus at 4 °C for 30 min. After washing with deionized water twice and treatment with ethanol (70%), the slides were dried and stained with the provided dye (50 μL). Images were captured under a fluorescence microscope using the Nikon E2000 Microscope system and the accompanying software (Nikon Corporation, Tokyo, Japan). The comet length of each cell was measured and calculated using the Comet A v.1.0 image analysis software (Cells Biolab, Inc., Beijing, China).

### RNA interference (siRNA)

Huh7 and HepG2 cells were transfected by TRPC6 siRNA, Twist siRNA, Hif1-α siRNA, H2A.X siRNA or negative control (NC) siRNA (Santa Cruz, CA, USA) at 25 nmol/L using Lipofectamine 3000 (Invitrogen), according to the manufacturer’s instructions. After siRNA transfection for 6 h, cells were cultured in complete medium for the designated time. The transfection efficiency was assessed by quantitative real-time reverse transcription PCR (qRT-PCR) and western blotting at 24 h and 48 h after transfection. All experiments were performed 24 h after siRNA transfection.

### Western blotting

HCC cells were seeded in 6-well plates at a density of 1.5 × 10^5^ cells per well and incubated for 12 h. After various treatments, the cells were washed with PBS twice and harvested immediately in ice-cold lysis buffer (150 μL) (Cell Signaling, Danvers, MA, USA). After the protein lysates were quantified using a Bicinchoninic Acid Protein Assay kit (Beyotime Institute of Biotechnology, Shanghai, China), protein solutions (40 μg/L) were separated using 10% SDS–PAGE and transferred electrophoretically to PVDF membranes (Millipore, Billerica, MA, USA). Membranes were blocked using 5% bovine serum albumin (BSA) in Tris-buffered saline (TBS) with 0.1% Tween-20 (TBST) for 1 h at room temperature and then incubated at 4 °C with anti-E-Cadherin, anti-Vimentin, anti-Claudin1, anti-phospho-ATM, anti-ATM, anti-phospho-ATR, anti-ATR, anti-phospho-H2A.X, anti-H2A.X, anti-phospho-Erk1/2 (Thr202/Tyr204), anti-Erk1/2, anti-phospho-AKT (Ser473), anti-AKT, anti-phospho-STAT3 (Tyr705), anti-STAT3 (Cell Signaling), anti-Hif1-α, anti-TRPC6 and anti-Twist (Abcam, Shanghai, China) antibodies, separately, at a dilution of 1:1000–2000, according to the manufacturer’s instructions. Protein loading was normalized using an anti-GAPDH antibody (1:5000, Kangchen Biotechnology, China). After incubating with the primary antibodies for 8h, membranes were then washed three times (10 min each) with TBST and incubated with the appropriate anti-mouse and anti-rabbit HRP-conjugated secondary antibodies (1:2000; GE Healthcare, Piscataway, NJ, USA). Images were developed using the ECL reagent (Millipore), and acquired using a VersaDoc Digital Imaging System (Bio-Rad).

### qRT-PCR

The total RNA of each sample was extracted and purified using the TRIzol reagent (Invitrogen), according to the manufacturer’s instructions. A PrimeScript RT reagent kit (Takara Biotechnology Co., Dalian, China) was used for reverse transcription of RNA (1 μg) to cDNA, which was amplified according to the following program: 95 °C for 30 s; then 40 cycles of 95 °C for 5 s and 60 °C for 34 s; 95 °C for 15 s; 60 °C for 1 min and 95 °C for 15 s. The procedure was realized in a StepOne Plus Real Time PCR instrument (Applied Biosystems, Carlsbad, CA, USA) using equal volumes of cDNA (2 μL) with the SYBR Premix Ex Taq II kit (Takara), according to the manufacturer’s instructions. Relative quantitation analysis was performed with reference to *β-actin* RNA using the comparative cycle threshold (CT) method. The primer sequences were listed as followed: TRPC6, Forward (5′-3′): GACATCTTCAAGTTCATGGTCACA, Reverse (5′-3′): ATCAGCGTCATCCTCAATTTC; β-actin, Forward (5′-3′): CGTGCGTGACATTAAAGAG, Reverse (5′-3′): TTGCCGATAGTGATGACCT.

### Construction and production of lentiviral vectors for stable TRPC6 silencing

The GV115 lentiviral particles containing short hairpin sequence targeting the human *TRPC6* gene (GenBank ID: NM_004621) as well as the negative sequence, were purchased from GeneChem (Shanghai, China). The sequences for the siRNA targeting TRPC6 gene and the non-silencing siRNA were as follows: TRPC6, TGGGTAATAGGCATGATAT; non-silencing siRNA, TTCTCCGAACGTGTCACGT. The oligonucleotides designed according to the structure of the siRNA were TRPC6 sense 5′-CCGGCCTGGGTAATAGGCATGATATCTCGAGATATCATGCCTATTACCCAGGTTTTTG-3′ and antisense 5′-AATTCAAAAACCTGGGTAATAGGCATGATATCTCGAGATATCATGCCTATTACCCAGG-3′, negative control sense 5′-CCGGTTCTCCGAACGTGTCACGTTTCAAGAGAACGTGACACGTTCGGAGAATTTTTG-3′ and antisense 5′-AATTCAAAAATTCTCCGAACGTGTCACGTTCTCTTGAAACGTGACACGTTCGGAGAA-3′. The oligonucleotides containing *TRPC6* siRNA or control siRNA sequences were constructed into plasmids, which were then cloned into the lentiviral vectors encoding a green fluorescent protein (GFP) sequence designated as LV-shTRPC6 and LV-NC. To generate the lentivirus, the recombinant vector and packaged plasmids were co-transduced into 293 T cells using lipofectamine 2000 (Invitrogen, USA). The final titre of the recombinant virus was 5 × 10^8^ TU/mL.

### HCC cells *in vivo* xenograft

This assay studied the effect of TRPC6 silencing on curative efficacy of doxorubicin to HCC *in vivo*. Huh7 cells were infected with lentivirus at MOI of 10 (LV-NC, LV shTRPC6), and 72 hours after infection, the infection efficiency was high enough with more than 90% of the cells expressing GFP. The cells (suspended in PBS, 1 × 10^6^ cells/100 μL) were injected subcutaneously into the right axillary fossa of twenty-four BALB/c athymic nude mice (Experiment Animal Centre, Shanghai, China), weighing 20–25g that were 3–4 weeks old (two groups for LV-NC, two groups for LV shTRPC6, n = 6 per group). Tumour length (L) and width (W) were measured every other day, and tumour volumes were calculated as follows: (L × W^2^)/2^58^. After the tumour volumes reached 100–150 mm^3^, half of the mice were intraperitoneally injected with doxorubicin (4 mg/kg in normal saline) and the other mice with the equal volume of normal saline. Doxorubicin was delivered intraperitoneally every two days for 2 weeks. After 2 weeks, mice were sacrificed by cervical dislocation, then tumours were resected and tumour weights were measured. The animal experiments were carried out in “accordance” with the approved guidelines of the Ethics Committee on Animal Experimentation of Zhejiang University, Hangzhou, China.

### Statistical analyses

Statistical analyses were performed using Prism 5 (GraphPad, San Diego, CA, USA). Statistical tests are detailed in the figure legends. For all experiments, p < 0.05 was considered statistically significant.

## Additional Information

**How to cite this article**: Wen, L. *et al.* Regulation of Multi-drug Resistance in hepatocellular carcinoma cells is TRPC6/Calcium Dependent. *Sci. Rep.* **6**, 23269; doi: 10.1038/srep23269 (2016).

## Supplementary Material

Supplementary Video 1

Supplementary Video 2

Supplementary Video 3

Supplementary Video 4

Supplementary Video 5

Supplementary Video 6

Supplementary Video 7

Supplementary Video 8

Supplementary Information

## Figures and Tables

**Table 1:** IC_50_ values of multiple drugs in HCC cells treated with doxorubicin (0.2 μg/mL) (Pre-Dox), hypoxia (1% O_2_), ionizing radiation (10 Gy) (Pre-IR), compared with control groups (normality) (n = 6).

Cell lines	Drugs	Stimuli			
		Normality	Hypoxia	Pre-Dox	Pre-IR
	Doxorubicin (μg/mL)	0.2026	1.062*	2.900*	1.130*
Huh7	5-Fluorouracil (mg/mL)	0.03799	0.2278*	3.106*	0.1739*
	Cisplatin (μg/mL)	2.831	5.841*	11.34*	5.054*
	Doxorubicin (μg/mL)	0.2776	0.5824*	1.311*	0.7392*
HepG2	5-Fluorouracil (mg/mL)	0.02016	0.05581*	1.282*	0.06292*
	Cisplatin (μg/mL)	3.288	6.630*	13.11*	5.937*

Statistical significances were assessed using Student’s T-Test.

^*^p < 0.05, stimuli groups compared with control groups (normality).

## References

[b1] Lage H. An overview of cancer multidrug resistance: a still unsolved problem. Cell Mol Life Sci 65, 3145–3167, doi: 10.1007/s00018-008-8111-5 (2008). 18581055 PMC11131739

[b2] Szakacs G., Paterson J. K., Ludwig J. A., Booth-Genthe C. & Gottesman M. M. Targeting multidrug resistance in cancer. Nat Rev Drug Discov 5, 219–234, doi: 10.1038/nrd1984 (2006). 16518375

[b3] Milane L., Ganesh S., Shah S., Duan Z. F. & Amiji M. Multi-modal strategies for overcoming tumor drug resistance: hypoxia, the Warburg effect, stem cells, and multifunctional nanotechnology. J Control Release 155, 237–247, doi: 10.1016/j.jconrel.2011.03.032 (2011). 21497176 PMC3146561

[b4] Wilson W. R. & Hay M. P. Targeting hypoxia in cancer therapy. Nat Rev Cancer 11, 393–410, doi: 10.1038/nrc3064 (2011). 21606941

[b5] Gottesman M. M. & Pastan I. Biochemistry of multidrug resistance mediated by the multidrug transporter. Annu Rev Biochem 62, 385–427, doi: 10.1146/annurev.bi.62.070193.002125 (1993). 8102521

[b6] Li Q. Q. *et al.* Twist1-mediated adriamycin-induced epithelial-mesenchymal transition relates to multidrug resistance and invasive potential in breast cancer cells. Clin Cancer Res 15, 2657–2665, doi: 10.1158/1078-0432.CCR-08-2372 (2009). 19336515

[b7] van Zijl F. *et al.* A human model of epithelial to mesenchymal transition to monitor drug efficacy in hepatocellular carcinoma progression. Mol Cancer Ther 10, 850–860, doi: 10.1158/1535-7163.MCT-10-0917 (2011). 21364009

[b8] Comerford K. M. *et al.* Hypoxia-inducible factor-1-dependent regulation of the multidrug resistance (MDR1) gene. Cancer Res 62, 3387–3394 (2002). 12067980

[b9] Park J. G. *et al.* MDR1 gene expression: its effect on drug resistance to doxorubicin in human hepatocellular carcinoma cell lines. J Natl Cancer Inst 86, 700–705 (1994). 7908989 10.1093/jnci/86.9.700

[b10] Venkatesha V. A. *et al.* Sensitization of pancreatic cancer stem cells to gemcitabine by Chk1 inhibition. Neoplasia 14, 519–525 (2012). 22787433 10.1593/neo.12538 PMC3394194

[b11] Wang L., Mosel A. J., Oakley G. G. & Peng A. Deficient DNA damage signaling leads to chemoresistance to cisplatin in oral cancer. Mol Cancer Ther 11, 2401–2409, doi: 10.1158/1535-7163.MCT-12-0448 (2012). 22973056 PMC3496048

[b12] Yang Z. J., Chee C. E., Huang S. & Sinicrope F. A. The role of autophagy in cancer: therapeutic implications. Mol Cancer Ther 10, 1533–1541, doi: 10.1158/1535-7163.MCT-11-0047 (2011). 21878654 PMC3170456

[b13] Martin V. *et al.* Melatonin-induced methylation of the ABCG2/BCRP promoter as a novel mechanism to overcome multidrug resistance in brain tumour stem cells. Br J Cancer 108, 2005–2012, doi: 10.1038/bjc.2013.188 (2013). 23632480 PMC3670480

[b14] Baguley B. C. Tumor stem cell niches: a new functional framework for the action of anticancer drugs. Recent Pat Anticancer Drug Discov 1, 121–127 (2006). 18221031 10.2174/157489206775246494

[b15] Wu Q., Yang Z., Nie Y., Shi Y. & Fan D. Multi-drug resistance in cancer chemotherapeutics: mechanisms and lab approaches. Cancer Lett 347, 159–166, doi: 10.1016/j.canlet.2014.03.013 (2014). 24657660

[b16] Teicher B. A. Acute and chronic *in vivo* therapeutic resistance. Biochem Pharmacol 77, 1665–1673, doi: 10.1016/j.bcp.2009.01.006 (2009). 19428320

[b17] Petraccia L. *et al.* [MDR (multidrug resistance) in hepatocarcinoma clinical-therapeutic implications]. Clin Ter 154, 325–335 (2003). 14994922

[b18] Luo D., Wang Z., Wu J., Jiang C. & Wu J. The role of hypoxia inducible factor-1 in hepatocellular carcinoma. Biomed Res Int 2014, 409272, doi: 10.1155/2014/409272 (2014). 25101278 PMC4101982

[b19] van Zijl F. *et al.* Epithelial-mesenchymal transition in hepatocellular carcinoma. Future Oncol 5, 1169–1179, doi: 10.2217/fon.09.91 (2009). 19852728 PMC2963061

[b20] Zhang H. *et al.* Targeting human 8-oxoguanine DNA glycosylase (hOGG1) to mitochondria enhances cisplatin cytotoxicity in hepatoma cells. Carcinogenesis 28, 1629–1637, doi: 10.1093/carcin/bgm072 (2007). 17389610

[b21] Berridge M. J., Bootman M. D. & Roderick H. L. Calcium signalling: dynamics, homeostasis and remodelling. Nat Rev Mol Cell Biol 4, 517–529, doi: 10.1038/nrm1155 (2003). 12838335

[b22] Hu J. *et al.* Downregulation of transcription factor Oct4 induces an epithelial-to-mesenchymal transition via enhancement of Ca^2+^ influx in breast cancer cells. Biochem Biophys Res Commun 411, 786–791, doi: 10.1016/j.bbrc.2011.07.025 (2011). 21798248

[b23] Davis F. M. *et al.* Non-stimulated, agonist-stimulated and store-operated Ca^2+^ influx in MDA-MB-468 breast cancer cells and the effect of EGF-induced EMT on calcium entry. PLoS One 7, e36923, doi: 10.1371/journal.pone.0036923 (2012). 22666335 PMC3364242

[b24] Davis F. M. *et al.* Induction of epithelial-mesenchymal transition (EMT) in breast cancer cells is calcium signal dependent. Oncogene 33, 2307–2316, doi: 10.1038/onc.2013.187 (2014). 23686305 PMC3917976

[b25] Mottet D. *et al.* ERK and calcium in activation of HIF-1. Ann N Y Acad Sci 973, 448–453 (2002). 12485909 10.1111/j.1749-6632.2002.tb04681.x

[b26] Mottet D. *et al.* Role of ERK and calcium in the hypoxia-induced activation of HIF-1. J Cell Physiol 194, 30–44, doi: 10.1002/jcp.10176 (2003). 12447987

[b27] Bentle M. S., Reinicke K. E., Bey E. A., Spitz D. R. & Boothman D. A. Calcium-dependent modulation of poly(ADP-ribose) polymerase-1 alters cellular metabolism and DNA repair. J Biol Chem 281, 33684–33696, doi: 10.1074/jbc.M603678200 (2006). 16920718

[b28] Monteith G. R., McAndrew D., Faddy H. M. & Roberts-Thomson S. J. Calcium and cancer: targeting Ca^2+^ transport. Nat Rev Cancer 7, 519–530, doi: 10.1038/nrc2171 (2007). 17585332

[b29] Chigurupati S. *et al.* Receptor channel TRPC6 is a key mediator of Notch-driven glioblastoma growth and invasiveness. Cancer Res 70, 418–427, doi: 10.1158/0008-5472.CAN-09-2654 (2010). 20028870

[b30] Ma X. *et al.* Transient receptor potential channel TRPC5 is essential for P-glycoprotein induction in drug-resistant cancer cells. Proc Natl Acad Sci USA 109, 16282–16287, doi: 10.1073/pnas.1202989109 (2012). 22988121 PMC3479621

[b31] Yu Y. *et al.* PDGF stimulates pulmonary vascular smooth muscle cell proliferation by upregulating TRPC6 expression. Am J Physiol Cell Physiol 284, C316–330, doi: 10.1152/ajpcell.00125.2002 (2003). 12529250

[b32] El Boustany C. *et al.* Capacitative calcium entry and transient receptor potential canonical 6 expression control human hepatoma cell proliferation. Hepatology 47, 2068–2077, doi: 10.1002/hep.22263 (2008). 18506892

[b33] Salnikow K. *et al.* Depletion of intracellular ascorbate by the carcinogenic metals nickel and cobalt results in the induction of hypoxic stress. J Biol Chem 279, 40337–40344, doi: 10.1074/jbc.M403057200 (2004). 15271983

[b34] Dongiovanni P. *et al.* Iron depletion by deferoxamine up-regulates glucose uptake and insulin signaling in hepatoma cells and in rat liver. Am J Pathol 172, 738–747, doi: 10.2353/ajpath.2008.070097 (2008). 18245813 PMC2258266

[b35] Berridge M. J., Lipp P. & Bootman M. D. The versatility and universality of calcium signalling. Nat Rev Mol Cell Biol 1, 11–21, doi: 10.1038/35036035 (2000). 11413485

[b36] Chen B. W. *et al.* Inhibition of mTORC2 Induces Cell-Cycle Arrest and Enhances the Cytotoxicity of Doxorubicin by Suppressing MDR1 Expression in HCC Cells. Mol Cancer Ther 14, 1805–1815, doi: 10.1158/1535-7163.MCT-15-0029 (2015). 26026051 PMC4866512

[b37] Hu Q. D. *et al.* NSC 74859 enhances doxorubicin cytotoxicity via inhibition of epithelial-mesenchymal transition in hepatocellular carcinoma cells. Cancer Lett 325, 207–213, doi: 10.1016/j.canlet.2012.07.003 (2012). 22781398

[b38] Celeste A. *et al.* Histone H2AX phosphorylation is dispensable for the initial recognition of DNA breaks. Nat Cell Biol 5, 675–679, doi: 10.1038/ncb1004 (2003). 12792649

[b39] Xu S. H. *et al.* ACK1 promotes gastric cancer epithelial-mesenchymal transition and metastasis through AKT-POU2F1-ECD signalling. J Pathol, doi: 10.1002/path.4515 (2015). 25678401

[b40] Li W. & Melton D. W. Cisplatin regulates the MAPK kinase pathway to induce increased expression of DNA repair gene ERCC1 and increase melanoma chemoresistance. Oncogene 31, 2412–2422, doi: 10.1038/onc.2011.426 (2012). 21996734

[b41] Zhao D. *et al.* Cytoplasmic p27 promotes epithelial-mesenchymal transition and tumor metastasis via STAT3-mediated Twist1 upregulation. Oncogene, doi: 10.1038/onc.2014.473 (2015). PMC4537852 25684140

[b42] Pawlus M. R., Wang L. & Hu C. J. STAT3 and HIF1alpha cooperatively activate HIF1 target genes in MDA-MB-231 and RCC4 cells. Oncogene 33, 1670–1679, doi: 10.1038/onc.2013.115 (2014). 23604114 PMC3868635

[b43] Liu W. L. *et al.* Targeting Phosphatidylinositide3-Kinase/Akt pathway by BKM120 for radiosensitization in hepatocellular carcinoma. Oncotarget 5, 3662–3672 (2014). 25004403 10.18632/oncotarget.1978 PMC4116511

[b44] Siddiquee K. *et al.* Selective chemical probe inhibitor of Stat3, identified through structure-based virtual screening, induces antitumor activity. Proc Natl Acad Sci USA 104, 7391–7396, doi: 10.1073/pnas.0609757104 (2007). 17463090 PMC1863497

[b45] Lin L. *et al.* The STAT3 inhibitor NSC 74859 is effective in hepatocellular cancers with disrupted TGF-beta signaling. Oncogene 28, 961–972, doi: 10.1038/onc.2008.448 (2009). 19137011 PMC2703464

[b46] Wang J. *et al.* Overexpression of von Hippel-Lindau protein synergizes with doxorubicin to suppress hepatocellular carcinoma in mice. J Hepatol 55, 359–368, doi: 10.1016/j.jhep.2010.10.043 (2011). 21168458

[b47] Tagliarino C. *et al.* Mu-calpain activation in beta-lapachone-mediated apoptosis. Cancer Biol Ther 2, 141–152 (2003). 12750552 10.4161/cbt.2.2.237

[b48] Sulova Z. *et al.* Does any relationship exist between P-glycoprotein-mediated multidrug resistance and intracellular calcium homeostasis. Gen Physiol Biophys 28 Spec No Focus, F89–95 (2009). 20093732

[b49] Yu Y. *et al.* Enhanced expression of transient receptor potential channels in idiopathic pulmonary arterial hypertension. Proc Natl Acad Sci USA 101, 13861–13866, doi: 10.1073/pnas.0405908101 (2004). 15358862 PMC518765

[b50] Ambudkar I. S. Ca^2+^ signaling microdomains:platforms for the assembly and regulation of TRPC channels. Trends Pharmacol Sci 27, 25–32, doi: 10.1016/j.tips.2005.11.008 (2006). 16337693

[b51] Minke B. TRP channels and Ca^2+^ signaling. Cell Calcium 40, 261–275, doi: 10.1016/j.ceca.2006.05.002 (2006). 16806461 PMC1934411

[b52] Bird G. S. *et al.* Mechanisms of phospholipase C-regulated calcium entry. Curr Mol Med 4, 291–301 (2004). 15101686 10.2174/1566524043360681

[b53] Iyer S. C., Kannan A., Gopal A., Devaraj N. & Halagowder D. Receptor channel TRPC6 orchestrate the activation of human hepatic stellate cell under hypoxia condition. Exp Cell Res 336, 66–75, doi: 10.1016/j.yexcr.2015.03.023 (2015). 25845497

[b54] Mahajan K. & Mahajan N. P. ACK1/TNK2 tyrosine kinase: molecular signaling and evolving role in cancers. Oncogene, doi: 10.1038/onc.2014.350 (2014). PMC4411206 25347744

[b55] Niu G. *et al.* Signal transducer and activator of transcription 3 is required for hypoxia-inducible factor-1alpha RNA expression in both tumor cells and tumor-associated myeloid cells. Mol Cancer Res 6, 1099–1105, doi: 10.1158/1541-7786.MCR-07-2177 (2008). 18644974 PMC2775817

[b56] Wendt M. K., Balanis N., Carlin C. R. & Schiemann W. P. STAT3 and epithelial-mesenchymal transitions in carcinomas. JAKSTAT 3, e28975, doi: 10.4161/jkst.28975 (2014). 24843831 PMC4024059

[b57] Courapied S. *et al.* The cdk5 kinase regulates the STAT3 transcription factor to prevent DNA damage upon topoisomerase I inhibition. J Biol Chem 285, 26765–26778, doi: 10.1074/jbc.M109.092304 (2010). 20516069 PMC2930675

[b58] Naito S., von Eschenbach A. C., Giavazzi R. & Fidler I. J. Growth and metastasis of tumor cells isolated from a human renal cell carcinoma implanted into different organs of nude mice. Cancer Res 46, 4109–4115 (1986). 3731078

