# Supplementary material for: Regulation of Multi-drug Resistance in hepatocellular carcinoma cells is TRPC6/Calcium Dependent
Source: Sci Rep. 2016 Mar 24;6:23269. doi: 10.1038/srep23269 (PMC4806320; doi:10.1038/srep23269)
Supplement: Supplementary Information [file srep23269-s9.doc]

Regulation of Multi-drug Resistance in hepatocellular carcinoma cells is TRPC6/Calcium Dependent

Running title: Calcium and Multi-drug Resistance in HCC cells

Liang Wen, Chao Liang, Enjiang Chen, Wei Chen, Feng Liang, Xiao Zhi, Tao Wei, Fei Xue, Guogang Li, Qi Yang, Weihua Gong, Xinhua Feng, Xueli Bai, Tingbo Liang

Contents

Supplemental Figure S1

Supplemental Figure S2

Supplemental Figure S3

Supplemental Figure S4

Supplemental Figure S5

Supplemental Figure S6

Supplemental Figure S7

Supplemental Figure S8

Supplemental Figure S9

Supplemental Figure S10

Supplemental Figure S11

Supplemental Figure S12

Supplemental Figure S13

Supplemental Figure S14

Supplemental Table S1

Supplemental Video S1

Supplemental Video S2

Supplemental Video S3

Supplemental Video S4

Supplemental Video S5

Supplemental Video S6

Supplemental Video S7

Supplemental Video S8

Supplementary Figure S1.The dose and duration of various stimuli are determined. (a) EMT induction occurs in the treatment of 0.2 μg/mL doxorubicin within 48 h. The expressions of epithelial phenotype markers, E-Cadherin and Claudin1, decrease and the mesenchymal phenotype marker, Vimentin, is upregulated during EMT (HepG2 cells express extremely low Vimentin). (b) Hif1-α induction occurs in the treatment of hypoxia within 72 h in HCC cells. Ionizing radiation (IR) of 10 Gy significantly enhanced HCC cells’ resistance to drugs. (c) 24 h after IR treatment of 0 Gy (normality), 3 Gy, 6 Gy or 10 Gy, HCC cells were then treated by doxorubicin (0.25 μg/mL), 5-Fluorouracil (40 μg/mL) and cisplatin (2 μg/mL) for 48 h, respectively. (*p<0.05, IR group *vs*. normality group). (d) 0, 2, 12 or 24 h after exposure to 10 Gy IR, HCC cells were then treated by doxorubicin (0.25 μg/mL) for 48 h. Statistical significances were assessed using Student’s T-Tests. (*p<0.05, “2 h after 10 Gy” group *vs*. “24 h after 10 Gy” group).


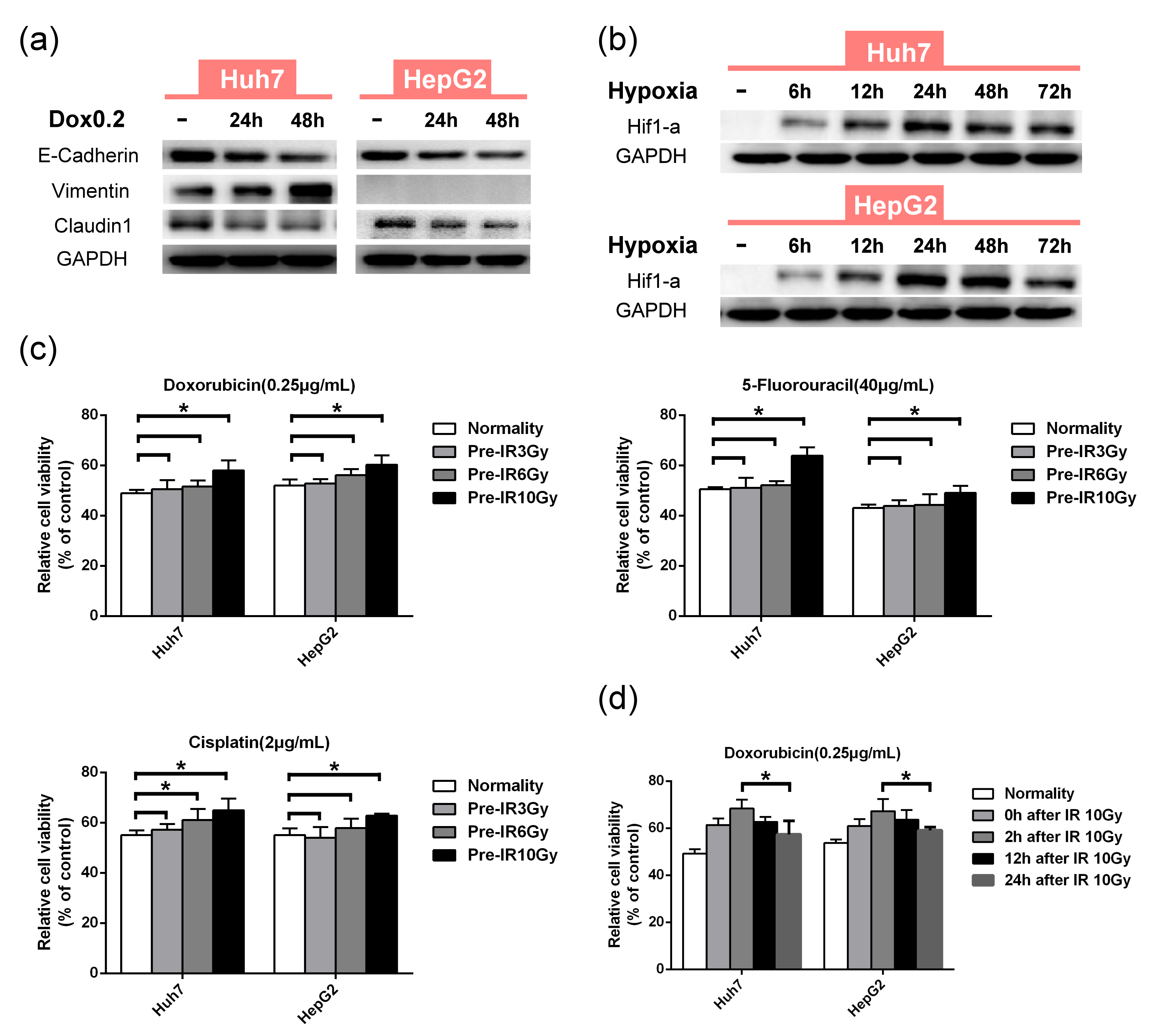


Supplementary Figure S2. Doxorubicin and deferoxamine fail to produce an immediate increase in cytosolic calcium in HepG2 cells. (a) Dynamic monitoring of [Ca^2+^]_c_ immunofluorescence within 30 min after adding doxorubicin (0.2 μg/mL), deferoxamine (100 μM), ionomycin (2 μg/mL) or HBSS (control group) to HepG2 cells incubated with Fluo4-AM calcium indicator. (b) Changes in mean cytosolic calcium fluorescence values of HepG2 cells (n=8-15) treated with doxorubicin (0.2 μg/mL) (blue), deferoxamine (100 μM) (green) and ionomycin (2 μg/mL) (red) are shown by the mean lines (solid) and 95%CI lines (dashed). Reagents were added at the 3 min time point. (c) The maximal calcium fluorescence values (mean ± SD) were calculated after adding doxorubicin (blue), deferoxamine (green) and ionomycin (red) within 30 min, respectively. Statistical significances were assessed using Student’s T-Test ( *p<0.05, each group *vs*. control group).


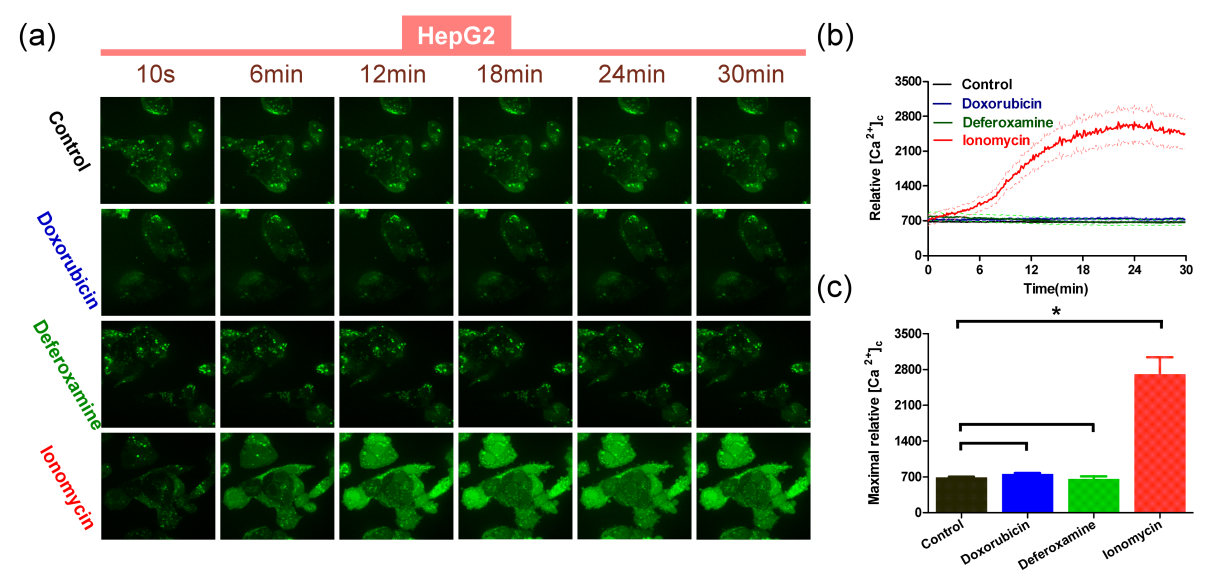


Supplementary Figure S3. Quantitative analysis of [Ca^2+^]_c_ positive cells. The results corresponding to those in Fig. 2d were repeated in three independent experiments, and the ratio (mean ± SD) of various stimuli-treated cells with higher calcium immunofluorescence intensities, compared with control groups, were calculated and presented. Statistical significances were assessed using Student’s T-Test (*p<0.05, each group *vs*. control group).


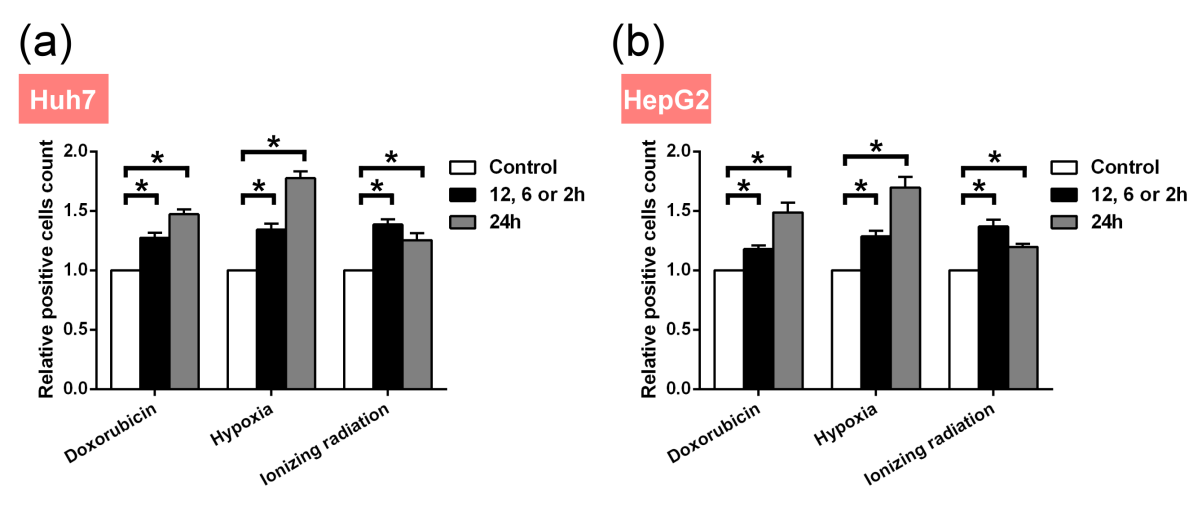


Supplementary Figure S4. Calcium chelation (BAPTA-AM, 10 μM) fails to affect the cell viability and drug sensitivity of HCC cells in normal conditions. (a) The relative cell viability (mean ± SD) (n=6) was tested 24 h after calcium chelation of different concentrations of BAPTA-AM. Statistical significances were assessed using Student’s T-Test. (*p<0.05, BAPTA-AM group *vs*. Control group).


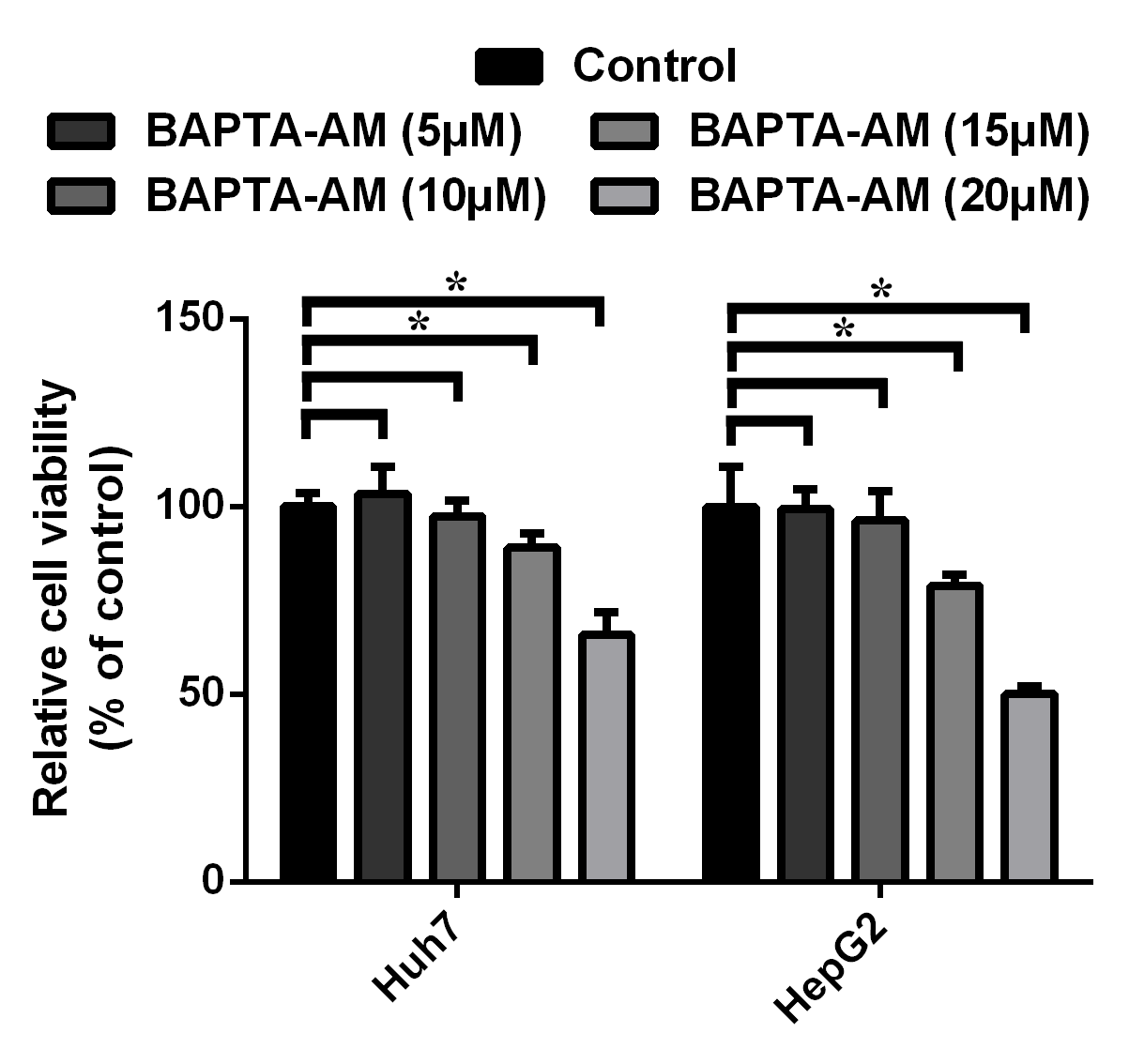


Supplementary Figure S5. Calcium chelation fails (p>0.05) to affecte the drug sensitivity of unstimulated cells (normality) to (a) 5-fluorouracil and (b) cisplatin but significantly (p<0.05) attenuates the enhancement of HCC cells’ resistance to (a) 5-fluorouracil and (b) cisplatin, in the stimuli of doxorubicin (0.2 μg/mL) (Pre-Dox) for 24 h, hypoxia (1% O_2_) for 6 h and ionizing radiation (10 Gy) (Pre-IR) (2 h later). Relative cell viability (Mean ± SD) (n=6) was calculated for control (black) groups and BAPTA-AM (orange) groups and best-fit lines are presented. Statistical significances were assessed using one-way ANOVA with Bonferroni’s post-tests (p<0.05, “Bapta-am+stimuli” group *vs*. stimuli group).


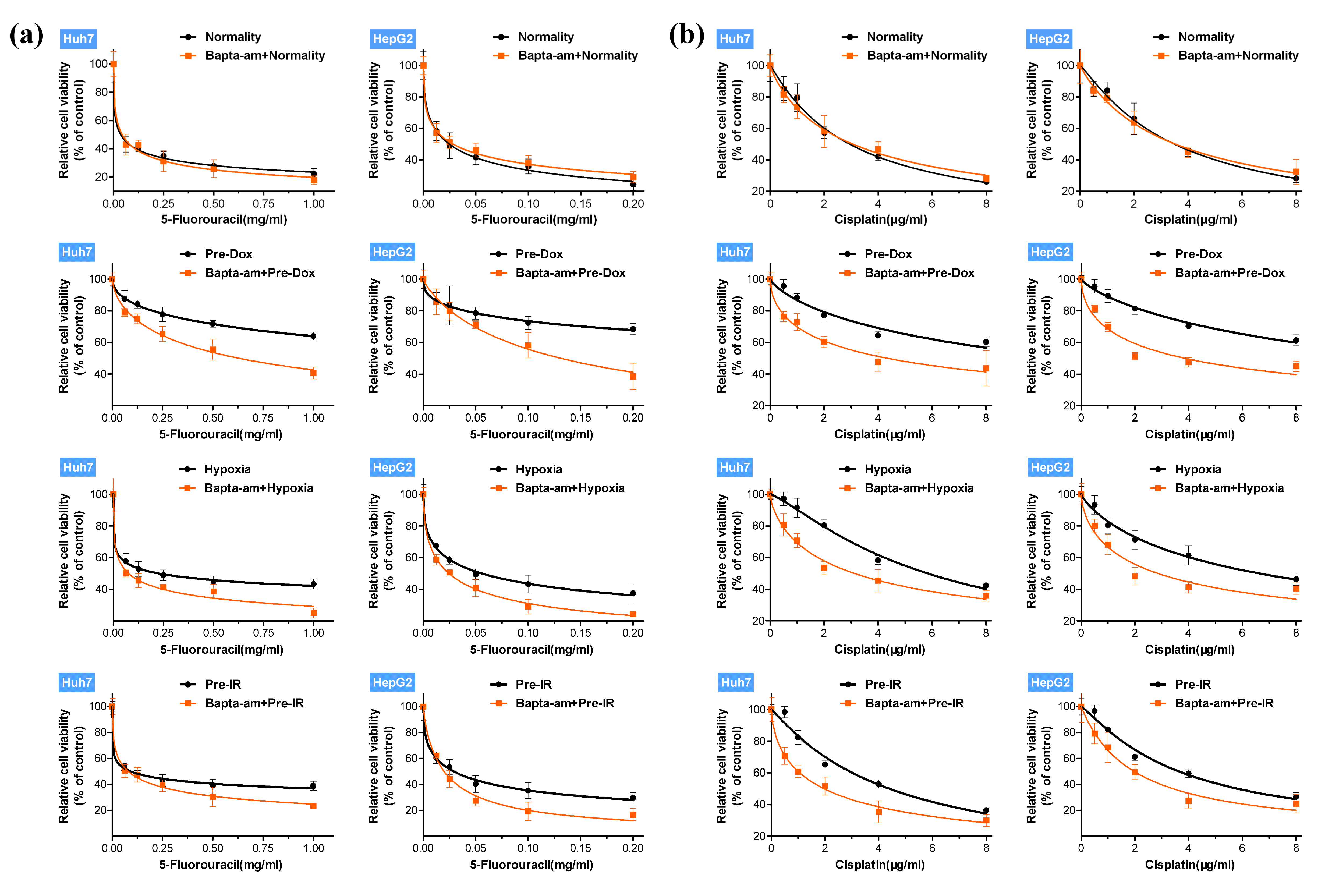


Supplementary Figure S6. Calcium chelation fails to inhibit the overexpression of ABCB1, ABCC1, ABCC2, ABCC3 or ABCG2 induced by doxorubicin (0.5 μg/mL). Relative expression of ABCB1，ABCC1，ABCC2，ABCC3 and ABCG2 mRNA (Mean ± SD) (n=3) after treatment with doxorubicin (0.5 μg/mL) for 24 h with or without calcium chelation (BAPTA-AM, 10 μM), compared with control groups, respectively, in Huh7 and HepG2 cells (*p<0.05, Dox0.5 group *vs*. control group, #p<0.05, “B10+Dox0.5” group *vs*. Dox0.5 group).


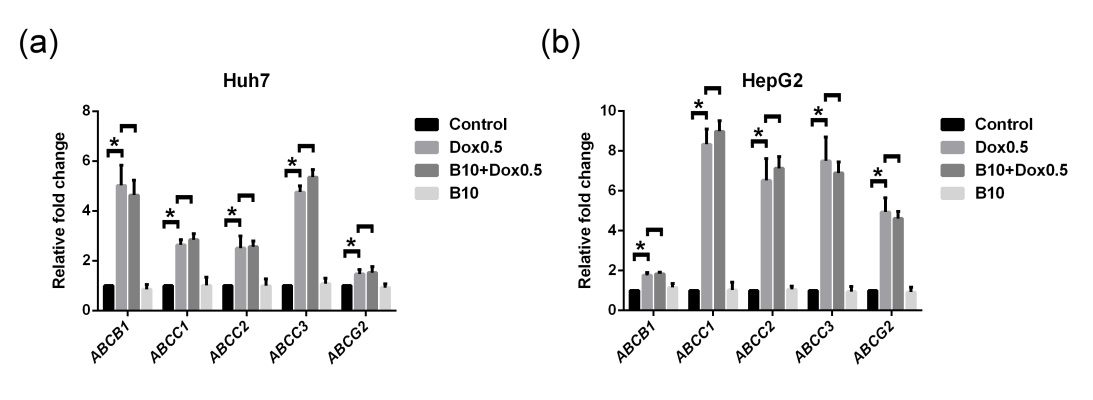


Supplementary Figure S7. Various siRNAs significantly attenuated multi-drug resistance induced by various stimuli, respectively. (a) Twist, Hif1-α and H2A.X protein expression 48 h after Twist, Hif1-α and H2A.X was regulated by siRNA (Si), respectively, compared with negative control (NC) (Hif1-α was expressed under hypoxia). Before stimuli of doxorubicin (Pre-Dox), hypoxia and ionizing radiation (Pre-IR), Huh7 and HepG2 cells were transfected with (b) Twist siRNA (siTwist), (c) Hif1-α siRNA (siHif1-α) and (d) H2A.X siRNA (siH2A.X), respectively. The relative cell viability (mean ± SD) (n=6) was tested by doxorubicin (1.0 μg/mL), 5-fluorouracil (Huh7, 0.5 mg/mL; HepG2, 0.1 mg/mL) or cisplatin (4.0 μg/mL). Statistical significances were assessed using Student’s T-Test. ( *p<0.05, siRNA group *vs*. NC group).


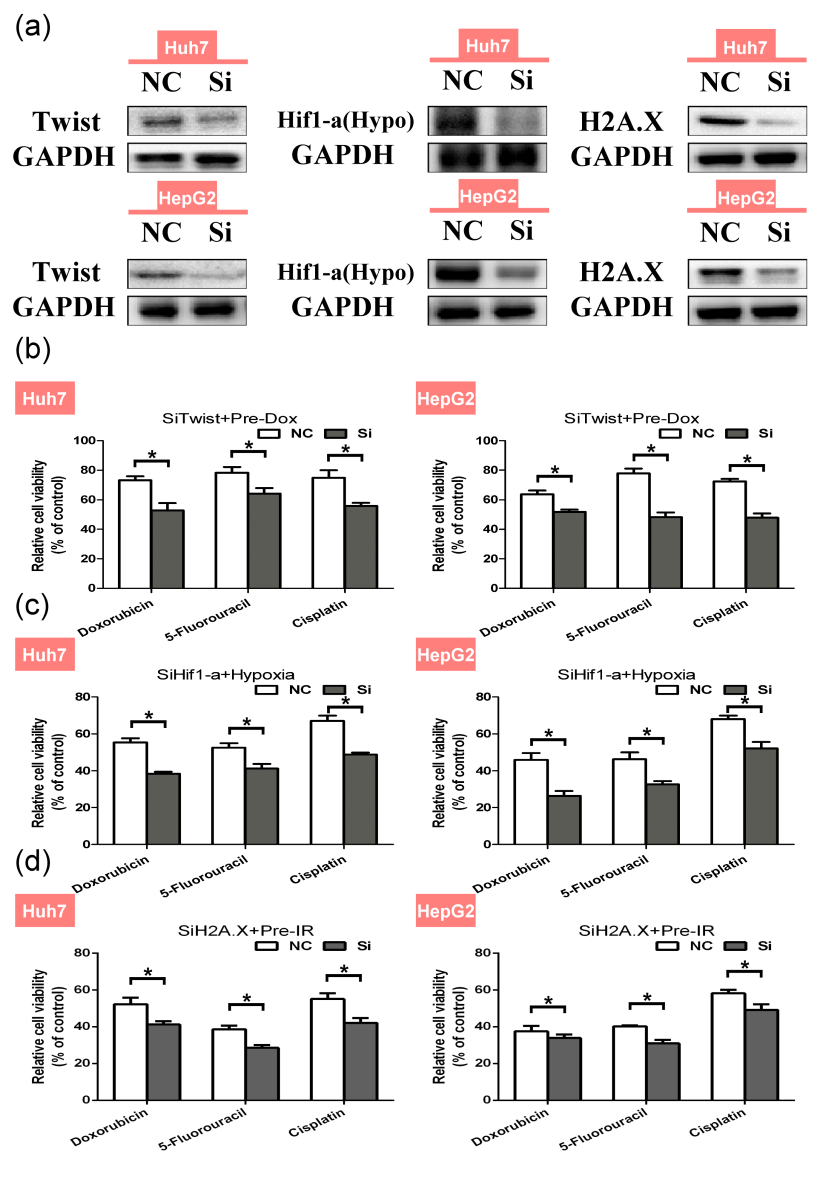


Supplementary Figure S8. (a) Calcium chelation inhibits the cellular transformation during EMT in HCC cells. Pretreatment with BAPTA-AM (10 μM) transformed the grape-like morphology induced by doxorubicin (Dox) (48 h) back to a spindle-like morphology, as observed under an optical microscope using a 40 × objective. (b) The total DNA was stained using a comet array and photographed 24 h after ionizing radiation (10Gy), with or without BAPTA-AM (10 μM) and (c) the relative comet lengths of cells (Mean ± SD) (n>6) in each group were measured in HCC cells. Statistical significances were assessed using Student’s T-Test. (*p<0.05, ionizing radiation (IR) group vs. control group; #p<0.05, “Bapta+IR” group *vs*. IR group).


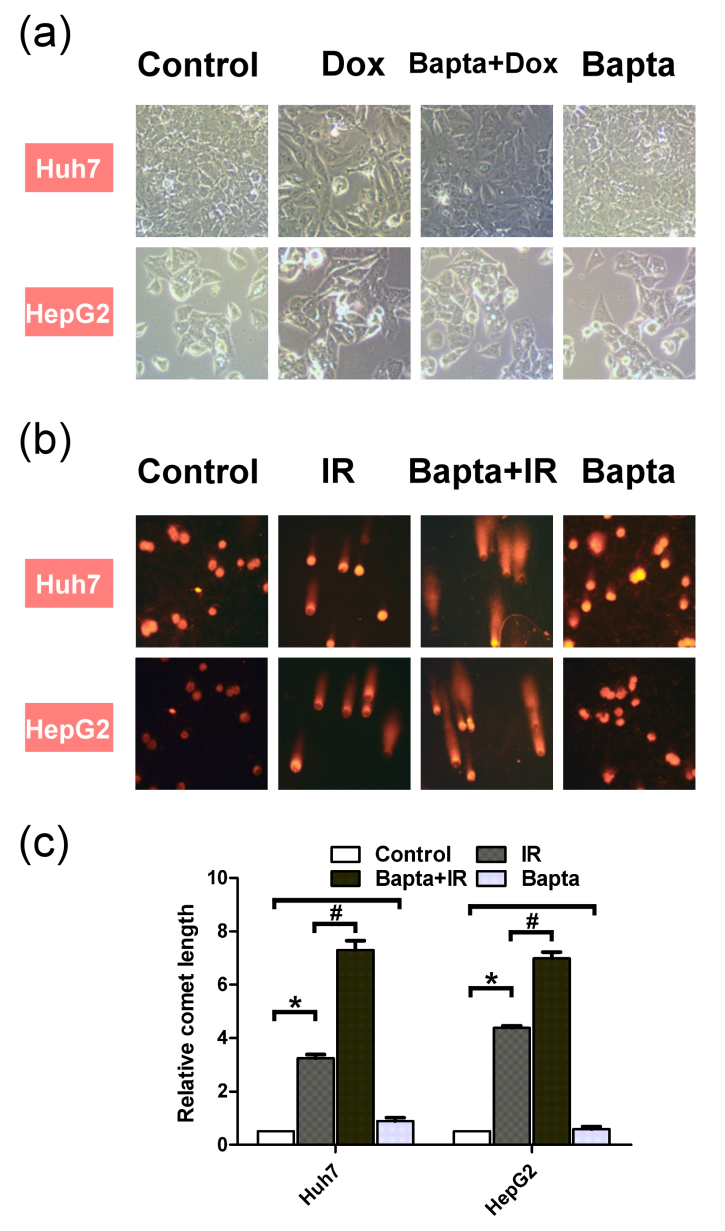


Supplementary Figure S9. Quantitative analysis of [Ca^2+^]_c_ positive. The results corresponding to those in Fig. 5b were repeated in three independent experiments, and the ratios (mean ± SD) of various stimuli-treated cells with higher calcium immunofluorescence intensity, compared with control groups, were calculated and presented. Statistical significances were assessed using Student’s T-Test. (*p<0.05, each group *vs*. control group; #p<0.05, each “SKF96365+stimuli” group *vs*. stimuli group).


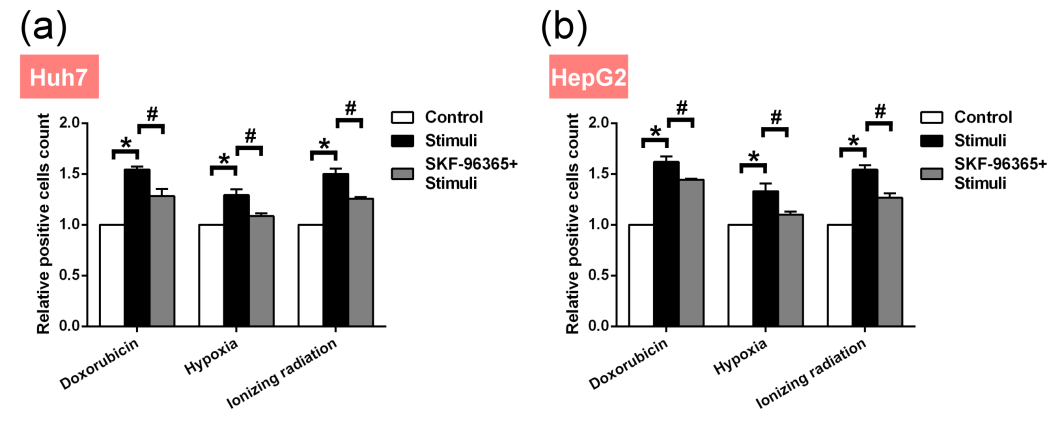


Supplementary Figure S10. Calcium channel TRPC6 expression after siRNA interference. (a) mRNA remaining 24 h after transfection with TRPC6 siRNA or negative control (NC) siRNA tested by qRT-PCR in six independent experiments. (*p<0.05, siTRPC6 group *vs*. NC group) (b) TRPC6 protein expression 48 h after TRPC6 interference (Si) compared with negative control (NC). Statistical significances were assessed using Student’s T-Test.


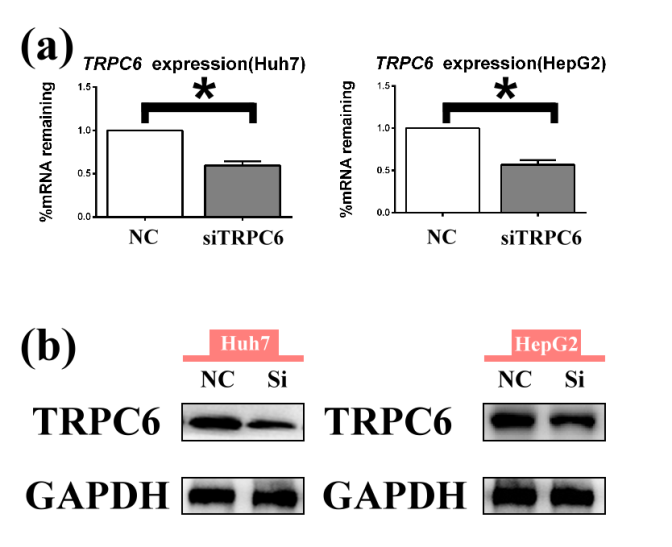


Supplementary Figure S11. TRPC6 interference significantly (p<0.05) attenuates enhancement of HCC cells’ resistance to (a) 5-fluorouracil and (b) cisplatin by stimuli of doxorubicin (Pre-Dox), hypoxia or ionizing radiation (Pre-IR). Relative cell viability (Mean ± SD) (n=6) was calculated for negative control (NC) (green) groups and TRPC6 siRNA (siTRPC6) (red) groups and best-fit lines are presented. Statistical significances were assessed using one-way ANOVA with Bonferroni’s post-tests (p<0.05, siTRPC6 group *vs*. NC group).


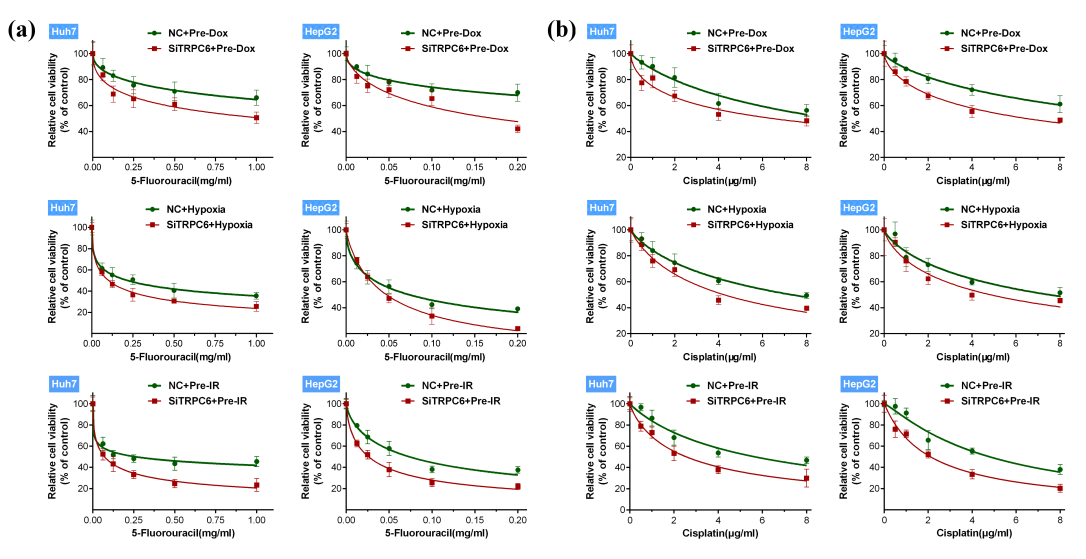


Supplementary Figure S12. Intracellular calcium chelation (a) enhances the expression of p-AKT (Ser473) and p-Erk (Thr202/Tyr204) but (b) inhibited p-STAT3 (Tyr705) induced by doxorubicin (0.2 μg/mL) (Dox) for 2 h, hypoxia (1% O_2_) (Hypo) for 2 h, and ionizing radiation (10 Gy) (IR) (2 h later) in Huh7 and HepG2 cells.


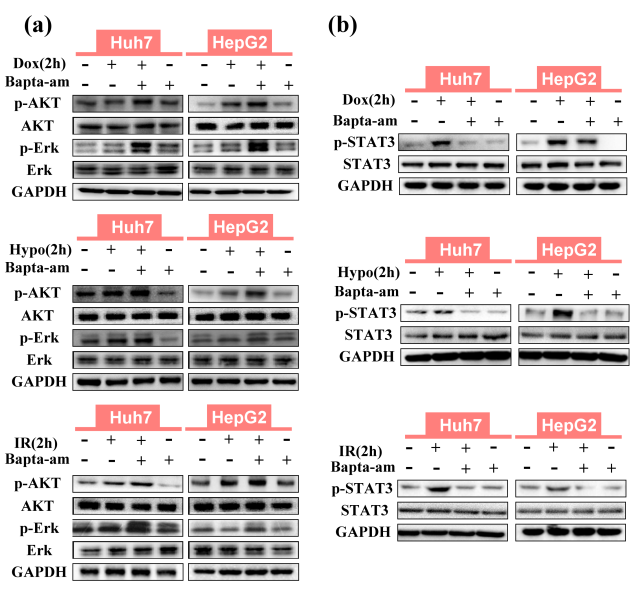


Supplementary Figure S13. Phosphorylation of STAT3 (Tyr705) is blocked by TRPC6 siRNA. Western blotting demonstrating the expression of p-STAT3 induced by doxorubicin (0.2 μg/mL) (Dox), hypoxia (1% O_2_) (Hypo) and ionizing radiation (10 Gy) (IR) at 24 h, 6 h and 2 h time point, respectively, with or without TRPC6 interference (SiTRPC6).


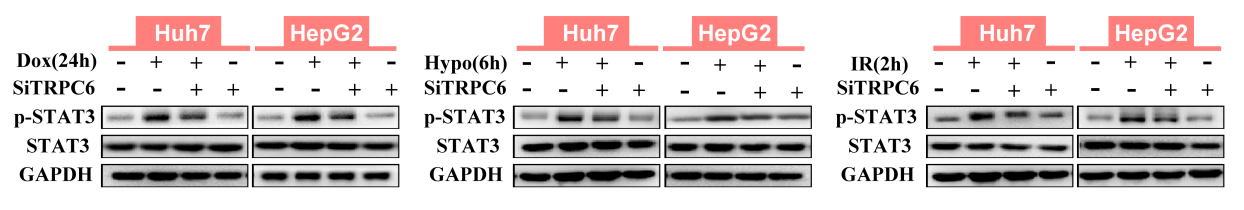


Supplementary Figure S14. STAT3 inhibitor NSC74859 (100 μM) significantly (p<0.05) attenuates the enhancement of HCC cells’ resistance to doxorubicin, treated by (a) doxorubicin (0.2 μg/mL) (Pre-Dox) for 24 h, (b) hypoxia (1% O_2_) for 6 h and (c) ionizing radiation (10 Gy) (Pre-IR) (2 h later). Relative cell viability (Mean ± SD) (n=6) was calculated with (grey) or without (black) NSC74859 and best-fit lines are presented. Statistical significances were assessed using one-way ANOVA with Bonferroni’s post-tests (p<0.05, NSC74859 group *vs*. control group).


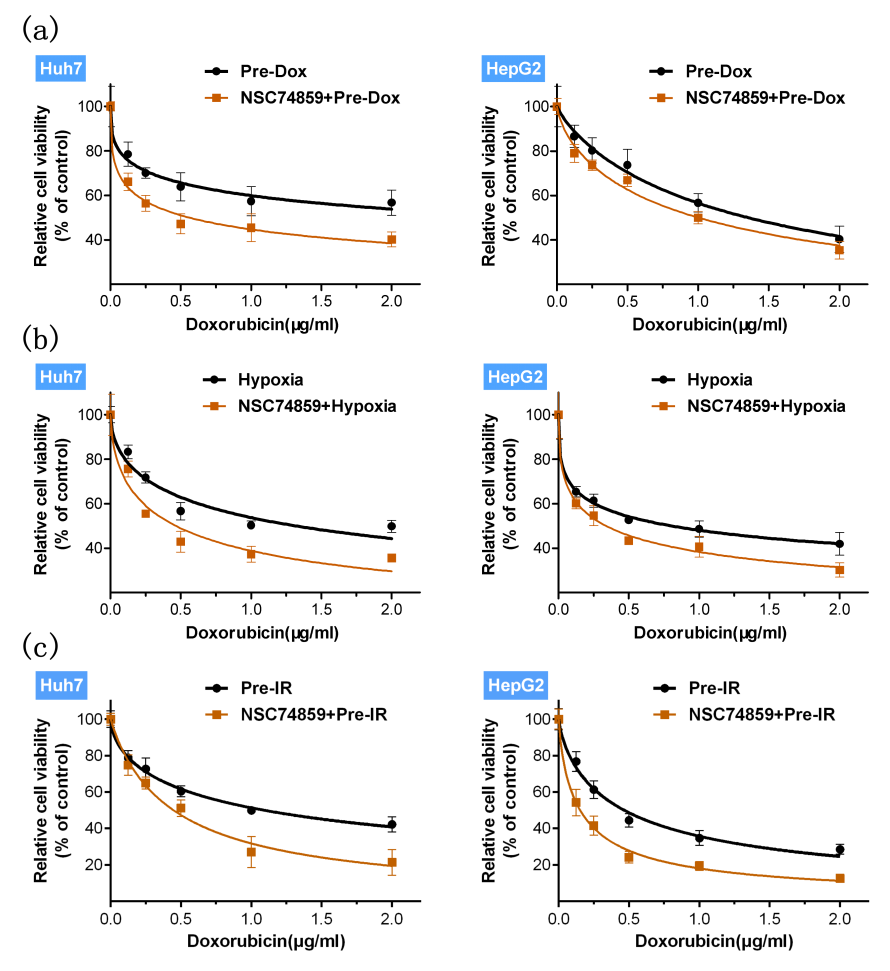


Table S1.

Pretreated with calcium chelation (BAPTA-AM, 10 μM), HCC cells’ drug resistance, induced by various stimuli (doxorubicin (Pre-Dox), hypoxia, ionizing radiation (Pre-IR) or normality), was assessed by IC_50_ values of multiple drugs (n=6).

| Cell lines | Drugs | Stimuli | | | |
| --- | --- | --- | --- | --- | --- |
|  |  | Normality | Hypoxia | Pre-Dox | Pre-IR |
|  | Doxorubicin(μg/mL) | 0.2443 | 0.5034* | 1.821* | 0.4101* |
| Huh7 | 5-Fluorouracil(mg/mL) | 0. 04334 | 0.07583* | 0.6308* | 0.08570* |
|  | Cisplatin (μg/mL) | 3.054 | 3.131 | 4.345* | 2.101* |
|  | Doxorubicin(μg/mL) | 0.3063 | 0.3414 | 0.5650* | 0.2352* |
| HepG2 | 5-Fluorouracil(mg/mL) | 0.02824 | 0.02450 | 0.1311* | 0.01857* |
|  | Cisplatin (μg/mL) | 3.524 | 2.864* | 3.863 | 2.541* |

Statistical significances were assessed using Student’s T-Test. *p<0.05, stimuli groups compared with unstimulated groups (normality).

Supplementary Video 1, 2, 3 and 4. Changes of intracellular calcium of Huh7 cells treated with HBSS, doxorubicin (0.2 μg/mL), deferoxamine (100 μM) and ionomycin (2 μg/mL) within 30 min, respectively. Reagents were added at the 3 min time point.

Supplementary Video 5, 6, 7 and 8. Changes of intracellular calcium of HepG2 cells treated with HBSS, doxorubicin (0.2 μg/mL), deferoxamine (100 μM) and ionomycin (2 μg/mL) within 30 min,, respectively. Reagents were added at the 3 min time point.
